# Systematic Access of Ternary Organotetrel‐Copper Chalcogenide Clusters by [PhTE_3_]^3−^ Anions (T=Si, Sn; E=S, Se)

**DOI:** 10.1002/chem.202101139

**Published:** 2021-06-22

**Authors:** Niklas Rinn, Lukas Guggolz, Han Yu Hou, Stefanie Dehnen

**Affiliations:** ^1^ Fachbereich Chemie Philipps Universität Marburg Hans-Meerwein Straße 4 35037 Marburg Germany

**Keywords:** copper, DFT studies, organotetrel chalcogenide clusters, UV/Vis spectroscopy, X-ray diffraction

## Abstract

Fragmentation reactions of organotetrel chalcogenide heteroadamantane‐type clusters [(PhT)_4_E_6_] (T/E=Si/S (**1**); Si/Se; Sn/S, and Sn/Se) by addition of the corresponding sodium chalcogenide gave salts of the general formula Na_3_[PhTE_3_], with T/E=Si/S (**2**); Si/Se (**3**); Sn/S (**A**); Sn/Se (**4**). Reaction of these salts with [Cu(PPh_3_)_3_Cl] gave a series of organotetrel–copper chalcogenide clusters [(CuPPh_3_)_6_(PhTE_3_)_2_] with T/E=Si/S; (**5**), Si/Se (**6**), Sn/S (**7**) and Sn/Se (**8**). Compounds **5**–**8** share a common structural motif with two intact {PhTE_3_} units coordinating a Cu_6_ moiety, which was previously reported with other ligands, and for the Sn and Ge congeners only. If the Sn/Se reaction system was allowed to crystallize more slowly, single crystals of compound [(CuPPh_3_)_6_(PhSnSe_3_)_3_Cu_3_SnSe] (**9**) were obtained, which are based on a larger cluster structure. Hence, **9** might form from **8** through incorporation of additional cluster fragments. The experimentally and quantum chemically determined optical properties were compared to related clusters.

## Graphical abstract

**Clusters of the type** [(CuPPh_2_Ph)_6_(PhTE_3_)_2_] (T=Si, Sn; E=S, Se) and [(CuPPh_3_)_6_(PhSnSe_3_)_3_Cu_3_SnSe] were formed in reactions of Na_3_[PhTE_3_] with [Cu(PPh_3_)_3_Cl]. The larger cluster based on T/E=Sn/Se clearly followed upon partial degradation of the [PhSnSe_3_]^3−^ subunit and exhibited a new cluster archetype with idealized *C*_3*v*_ symmetry. Quantum chemical studies served to shed light on the bonding situation in these clusters and to rationalize the optical absorption properties measured by solid‐state UV/Vis absorption spectra.

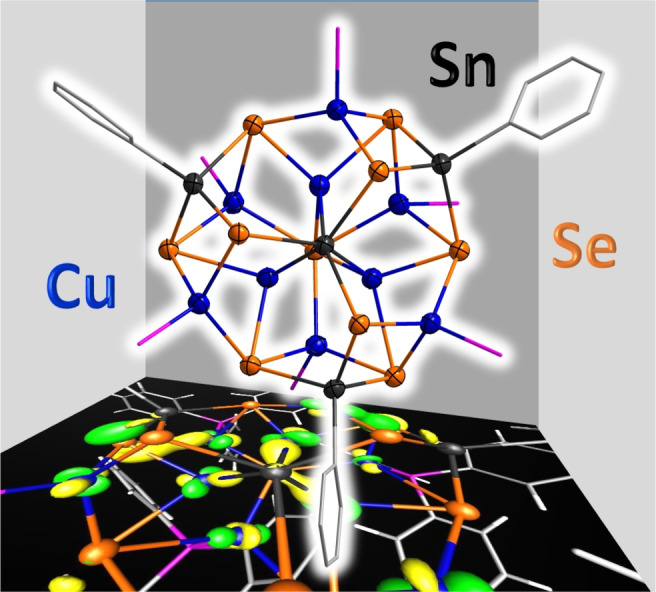

## Introduction

Ternary chalcogenide compounds of copper and a p‐block metal, in particular CuSn(S/Se)‐based materials, are currently actively investigated because of their opto‐electronic as well as their thermoelectric properties.[^1^, ^2^] Discrete complexes and clusters of such compositions have received a lot of attention in recent times owing to their potential as precursors to said materials, as showcased for CuInE_2_ (E=S, Se), a promising semiconductor material for energy conversion in solar cells.[^3^, ^4^, ^5^, ^6^, ^7^] For this reason, many activities in this direction so far have addressed group 13 compounds, be it as supertetrahedral anions[^8^, ^9^, ^10^, ^11^, ^12^] or as neutral cages.[^13^, ^14^, ^15^, ^16^, ^17^] Krautscheid and co‐authors have published a series of investigations on compounds exhibiting organic substituents at the group 13 metal, such as [R_3_PCu)_4_(MeM)_4_E_6_] (R=organic substituent; M=Ga, In).[^18^, ^19^, ^20^, ^21^] These studies were also expanded to Al compounds like [^i^Pr_3_PCuSC_2_H_4_SAlR_2_]_2_.[^22^, ^23^, ^24^]

While only a few species exist for related compounds including group 15 (semi)metals,[^25^, ^26^, ^27^] corresponding tin clusters have been explored to a much greater extent. As with the group 13 clusters, tin containing T5‐type supertetrahedral anions, e. g., [Cu_2_Ga_16_Sn_2_S_35_],^[28]^ as well as cages with ligand‐free tin atoms, like [Cu_6_Sn_6_S_20_]^10−^,[^29^, ^30^, ^31^] have been reported. Notably, there is also one study about analogous Pb complexes [{(PPh_3_)_3_}Cu_5_(μ‐SPh)_7_Pb] and [Pb(μ‐SPh)_4_{Cu(PPh_3_)_2_}_2_].^[32]^ Regarding organo‐functionalized clusters, a synthesis from mononuclear compounds is possible,^[33]^ but most of the reported clusters were synthesized by transformations of binary cage compounds of the general formulas [(RSn)_4_E_6_] or [(RSn)_3_E_4_Cl] (R=organic substituent, E=S, Se).[^34^, ^35^, ^36^, ^37^, ^38^, ^39^, ^40^] One of the first compounds of this type, [(CuPPhMe_2_)_6_(PhSnS_3_)_2_],^[40]^ was synthesized by using [Cu(PPhMe_2_)bipyCl] (bipy=2,2’‐bipyridine) as the source of Cu atoms, while fragmentation of the heteroadamantane compound [(PhSn)_4_S_6_] with Na_2_S formed [PhSnS_3_]^3−^ in situ, as the source of the RSn units.^[41]^ This was the first example of the inorganic cluster core archetype [(CuPPh_3‐x_R′_x_)_6_(RTE_3_)_2_] (x=0, 1, 2), consisting of six Cu atoms arranged in a chair conformation that is connected to two TE_3_ groups to obtain an overall spherical architecture. A systematic study of the three chalcogenide homologues of [(CuPPh_2_Et)_6_(PhSnE_3_)_2_] (E=S, Se, Te) that focused on the optical properties of such multinary clusters was recently reported by Eichhöfer et al.^[42]^

We are interested in the whole series of compounds of the general type [(RT)_4_E_6_] (T=Si, Ge, Sn; E=S, Se) for their peculiar non‐linear optical properties[^43^, ^44^, ^45^, ^46^] and also to address their post‐synthetic expansion with regard to both the organic shell and the inorganic cluster core.^[47]^ The ultimate goal of the project is providing a series of clusters based on ternary Cu/T/E architectures that may serve as precursors to thermal decomposition processes. We also introduced new functional substituents, such as ketones, carboxylic acids, as well as amino acid derivatives and peptides.^[38]^ The synthetic protocol was expanded for clusters comprising coinage metal atoms to the Ge congeners and to corresponding Ag and Au compounds.[^34^, ^36^, ^37^, ^38^, ^48^, ^49^, ^50^, ^51^, ^52^, ^53^] A combined organic‐inorganic extension of the substituents was possible by decoration of clusters with metallocenes,[^49^, ^54^, ^55^, ^56^, ^57^, ^58^, ^59^] or with chelate ligands to trap transition metals from solution.^[60]^ To the best of our knowledge, corresponding ternary Si clusters have not yet been reported in the literature. Indeed, the only reported complex with an Si/E/Cu inorganic core is [Cu_2_{(SSiMe_2_)_2_S}(PEt_3_)_3_].^[61]^ Moreover, in none of the studies regarding cluster re‐organization upon transition metal inclusion, [RTE_3_]^3−^ intermediates were isolated prior to use, but their presence in the reaction solutions was suspected, as the species have been reported in other contexts. To systematically vary and expand the described investigations we expanded the library of heteroadamantane‐type clusters to the heretofore unknown Si/Se congeners. Upon isolation and characterization of [(PhSi)_4_Se_6_] (**1**), we synthesized various reactants of the type Na_3_[PhTE_3_], namely Na_3_[PhSiS_3_] (**2**), Na_3_[PhSiSe_3_] (**3**), Na_3_[PhSnS_3_] (**A**, previously reported),^[41]^ and Na_3_[PhSnSe_3_] (**4**), and reacted them with [Cu(PPh_3_)_3_Cl]. This led to the formation of different types of ternary organotetrel copper chalcogenide clusters, the properties of which are presented and discussed in this article.

## Results and Discussion

### Syntheses

**Scheme 1:**
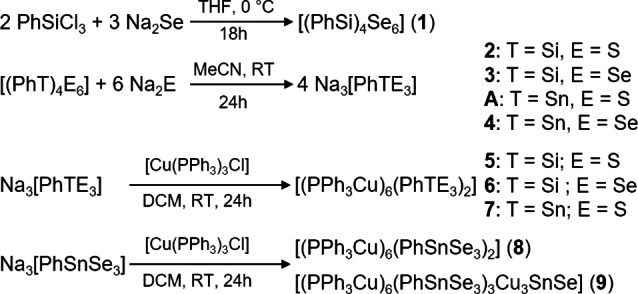
Reaction schemes from heteroadamantanes via Na_3_[PhTE_3_] species towards ternary copper compounds.

**Scheme 2:**
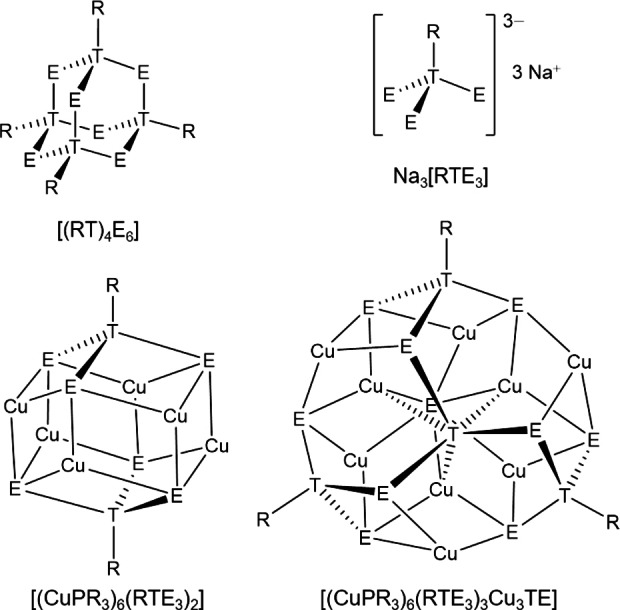
Diagrams of the structural motives discussed in this publication thereby emphasizing the coordination geometry at the tetrel atoms (T=Si, Sn) by E=S, Se, and Cu atoms. Phosphine ligands are omitted for clarity.

Compounds **2**, **3**, **A**, and **4** were prepared by reacting heteroadamantane‐type cages [(PhT)_4_E_6_], including the heretofore unknown [(PhSi)_4_Se_6_] (**1**), with Na_2_S or Na_2_Se respectively, in THF. By addition of [Cu(PPh_3_)_3_Cl] to **2**, **3**, or **A** in dichloromethane (DCM), we obtained the expected series of cluster compounds [(CuPPh_3_)_6_(PhSiS_3_)_2_] (**5**), [(CuPPh_3_)_6_(PhSiSe_3_)_2_] (**6**), and [(CuPPh_3_)_6_(PhSnS_3_)_2_] (**7**) after filtration of the reactive solutions and layering with *n*‐hexane. **5**–**7** share their common cluster core architecture with that of known compounds [(CuPPhMe_2_)_6_(PhSnS_3_)_2_]^[40]^ and [(CuPPh_3_)_6_(R^CO^GeS_3_)_2_] (R^CO^=Me(O)CCH_2_CMe_2_),^[38]^ both which were obtained in one‐pot reactions starting out from the respective heteroadamantane‐type cages, without isolation and clarification of the starting material. From the corresponding reaction of **4** with the Cu(I) source, however, two different cluster compounds can be isolated depending on the crystallization conditions. Compound [(CuPPh_3_)_6_(PhSnSe_3_)_2_] (**8**) crystallizes from a more concentrated filtrate within 24 hours, while layering of a more diluted solution with *n*‐hexane selectively leads to the formation of single crystals of [(CuPPh_3_)_6_(PhSnSe_3_)_3_Cu_3_SnSe] (**9**) within one week. **9** exhibits an extended cluster core with idealized *C*_3v_ symmetry and signs of a partial degradation of the [PhSnSe_3_]^3−^ moiety. A survey of the syntheses is given in Scheme 1. Diagrams of the structural motifs are shown in Scheme 2.

The different times for crystallization of **8** and **9** suggests that **9** results from incorporation of further Se, Sn and Cu fragments into the cluster core of **8**. These fragments might stem from partial decomposition of **8** or from left‐over starting material. The reason why this observation is made for the Sn/Se compounds only may lay in the difference in stability of the [PhSnSe_3_]^3−^, [PhSnS_3_]^3−^, and [PhSiE_3_]^3−^ units, hence causing Sn/Se fragments to form more readily in the reactive solution with time.

### Crystal structures and quantum chemical considerations

Crystallographic details of the new compounds are given in the Supporting Information, along with the structural descriptions of new solvates of known compound **A** and those of additional solvates of the new compounds, **5** ⋅ 2.76 DCM and **9** ⋅ 3.35 DCM, as the structures of the main molecules therein do not deviate from those reported here.^[62]^

**Figure 1:**
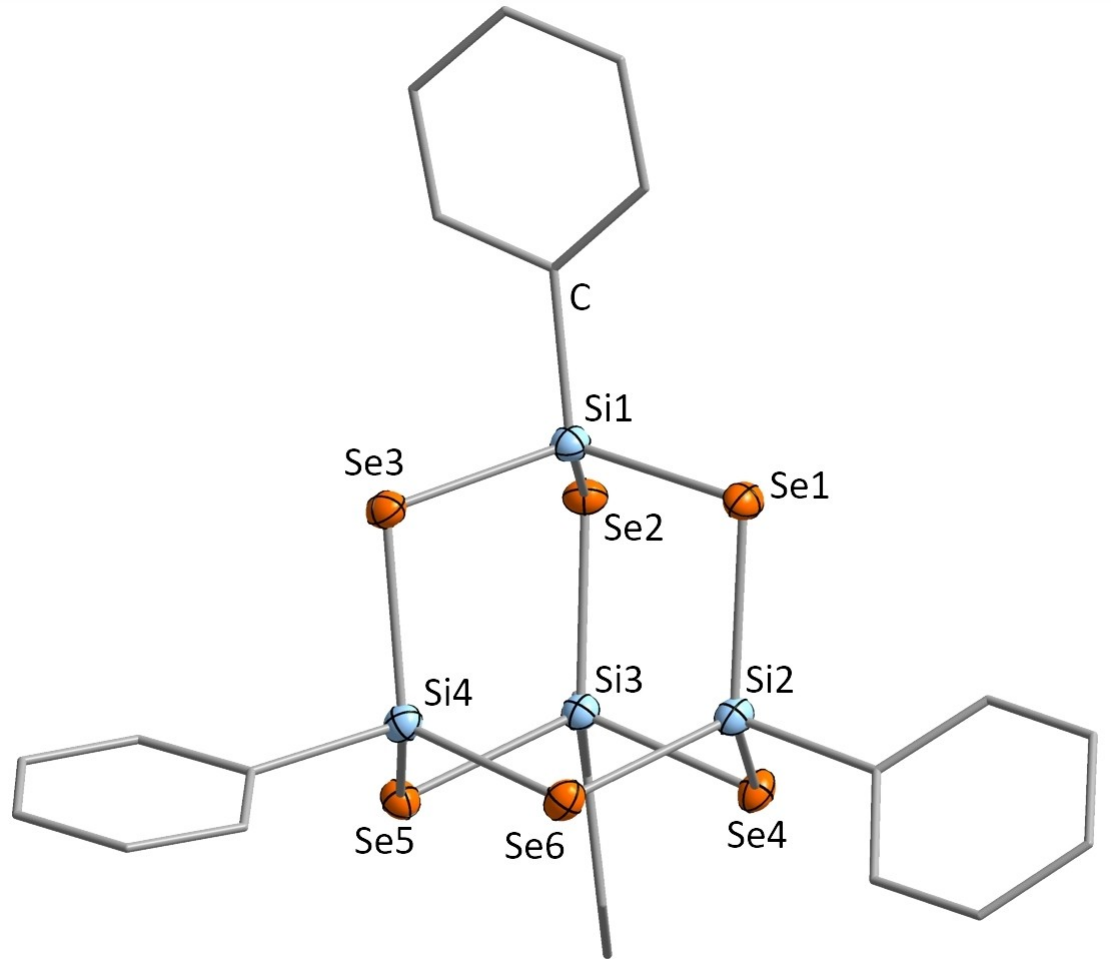
Molecular structure of compound **1**. Displacement ellipsoids are shown at 50 % probability; H atoms are omitted for clarity. Relevant distances and angles are mentioned in the text.

Compound **1**, comprising the heteroadamantane‐type cluster [(PhSi)_4_Se_6_], crystallizes in the monoclinic space group *P*2_1_/*c* and exhibits the expected molecular structure (Figure 1). However, the packing of the molecules in the crystal is not equivalent to that reported for its known sulfide congener.^[44]^ Additionally, we observe a slight irregularity, as two of the phenyl substituents are bent away from the Si−C axis: Si−C_ipso_‐C_para_=173.6(1)° (Si2) and 173.5(1)° (Si4) vs 178.0(1)° Si(1) and 177.6(1)° (Si3). We ascribe this finding to intermolecular interactions that seem to be stronger here than for the homologous compounds.

**Figure 2:**
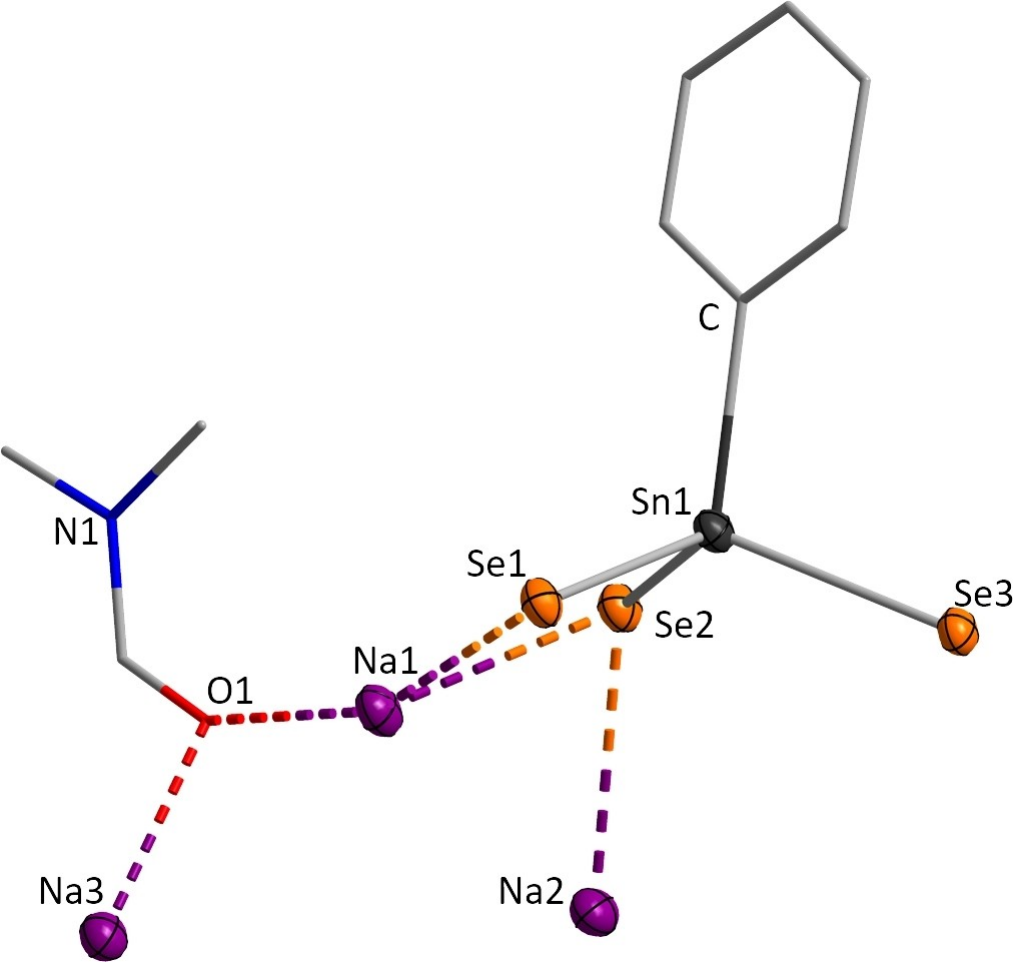
Asymmetric unit of the crystal structure of compound **4** ⋅ DMF. Displacement ellipsoids are shown at 50 % probability; H atoms are omitted for clarity. Selected distances and angles: Sn1‐Se 2.4876(4)–2.5270(5) Å; Se−Sn‐Se 107.87(2)–114.83(2)°.

While the silicon compounds **2** and **3** were obtained as powders, compound **4** ⋅ DMF was found to crystallize in the triclinic space group $P\bar{1}$ , hence representing the second crystalline compound of this series besides **A**. In the salt, layers of [PhSnSe_3_]^3−^ anions are connected by DMF‐bridged sodium atoms. This yields a coordination number of 5 for Na1 and Na2 and an octahedral coordination geometry for Na3. The asymmetric unit of **4** is shown in Figure 2.

**Figure 3:**
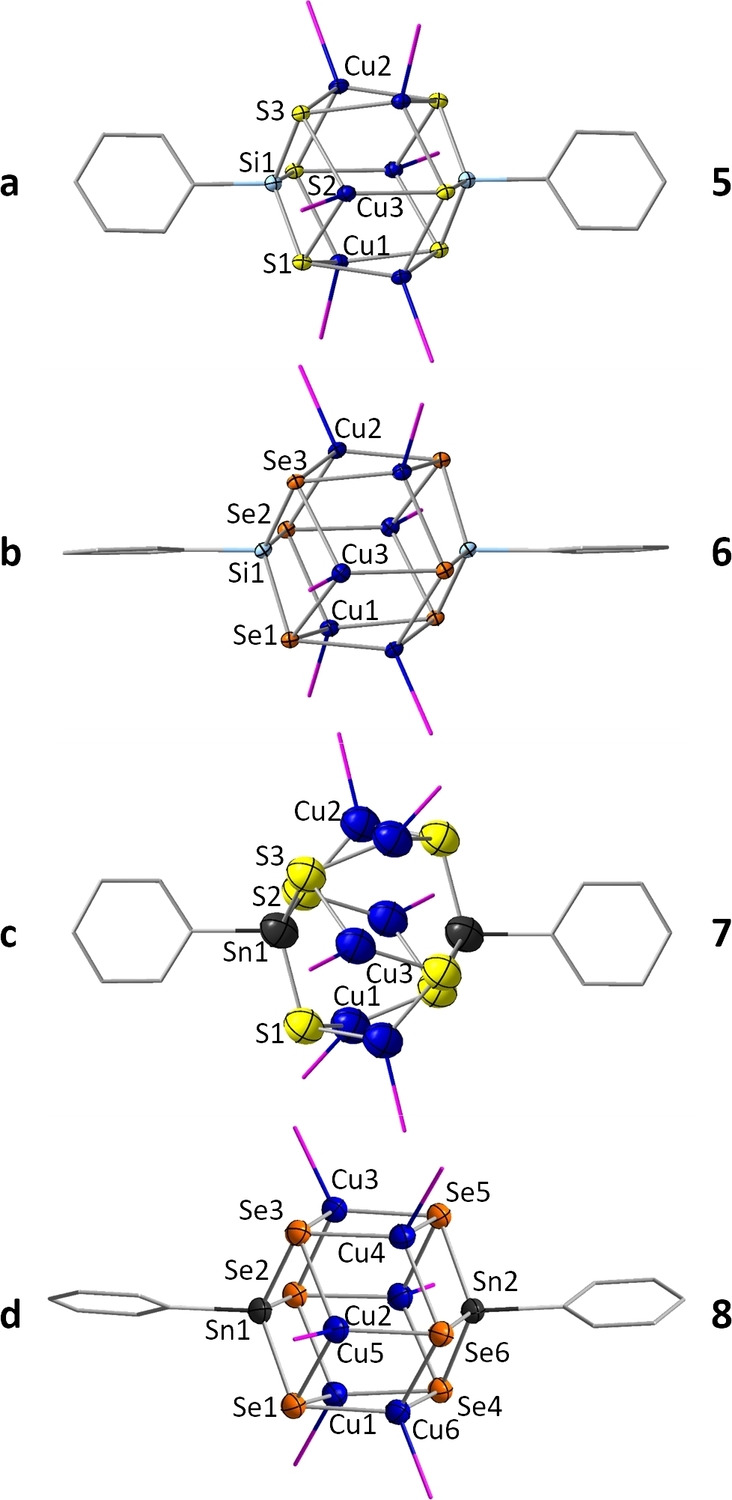
Molecular structure of **5** (a), **6** (b), **7** (c), and **8** (d). Displacement ellipsoids are shown at 50 % probability, H atoms and phenyl groups of the PPh_3_ ligands are omitted for clarity. P and C atoms are drawn as wires. Relevant structural parameters are mentioned in the text and detailed in the Supporting Information. Note that crystals of compound **7**, and to a lesser extend also of **8**, are notoriously of very poor quality, which we attribute to general differences of Sn/E cluster cores from Si/E cluster cores (see also below). The data set provided represents the best one we could get in many attempts (and different ways) to grow crystals.

Compounds **5**, **6**, **7**, and **8** are obtained in single crystalline form from reactions of the precursor compounds **2**, **3**, or **A**, respectively, with [Cu(PPh_3_)_3_Cl]. All of them are based on the same type of a ternary cluster core. Compounds **5** and **6** represent the first solely Si‐based copper organotetrel chalcogenide clusters. The clusters in **5** ⋅ 2 DCM (T/E=Si/S; monoclinic space group *P*2_1_/*c*; Figure 3a), and in **6** (T/E=Si/Se; triclinic space group $P\bar{1}$ ; Figure 3b) possess highly symmetric cluster cores, with idealized *S*_3_ symmetry that is intrinsically disturbed by the Si‐bonded phenyl groups. The high regularity of the Si_2_Cu_6_Se_6_ cluster core is evident by a relatively narrow range of Cu⋅⋅⋅Cu distances (2.6507(8)–2.8602(7) Å in compound **5**, 2.7716(6)–2.8827(6) Å in **6**).

The cluster in compound **7** (T/E=Sn/S; Figure 3c) differs by its phosphine ligands only from the reported, prototypic cluster [(CuPPhMe_2_)_6_(PhSnS_3_)_2_].^[40]^ It possesses a lower molecular symmetry than the two lighter congeners, which is reflected in a broader range of Cu⋅⋅⋅Cu distances (2.713(2)–3.278(3) Å) Furthermore, as can be taken from significantly larger thermal displacement parameters of the atoms than observed for the analogous compounds, compound **7** has only little tendency to crystallize. Despite many attempts, all crystals obtained of compound **7** were of poor quality. However, given that many examples of this class of compounds, in particular those with the general formula [(RT)_4_E_6_] (T=Si, Ge, Sn; E=S, Se) and R being an aromatic ligand, do not crystallize at all,[^43^, ^44^, ^45^, ^46^] we seem to have obtained a borderline case with this inorganic derivative.

Crystals of **8** ⋅ 4.4 DCM (space group *P*2_1_ */c*; Figure 3d) are not obtained by layering with *n*‐hexane, but crystallize – most likely as a consequence of oversaturation – from a more concentrated reactive solution (30 % volume) within one day after filtration. The cluster core found in **8** only exhibits a minor deviation from idealized *S*_3_ symmetry with Cu⋅⋅⋅Cu distances of 2.7822(8)–3.0653(8) Å, which are slightly larger than those in **5** and **6**. Layering with n‐hexane seems to inhibit crystallization of this compound. Hence, **8** can no longer be isolated, but instead, we observe the crystallization of **9**. Obviously, the stability of the [(CuPR’_3_)_6_(RTE_3_)_2_] cluster core in the reactive solution is limited, although its abundance in many different R’/RT/E combinations is remarkable.

**Figure 4:**
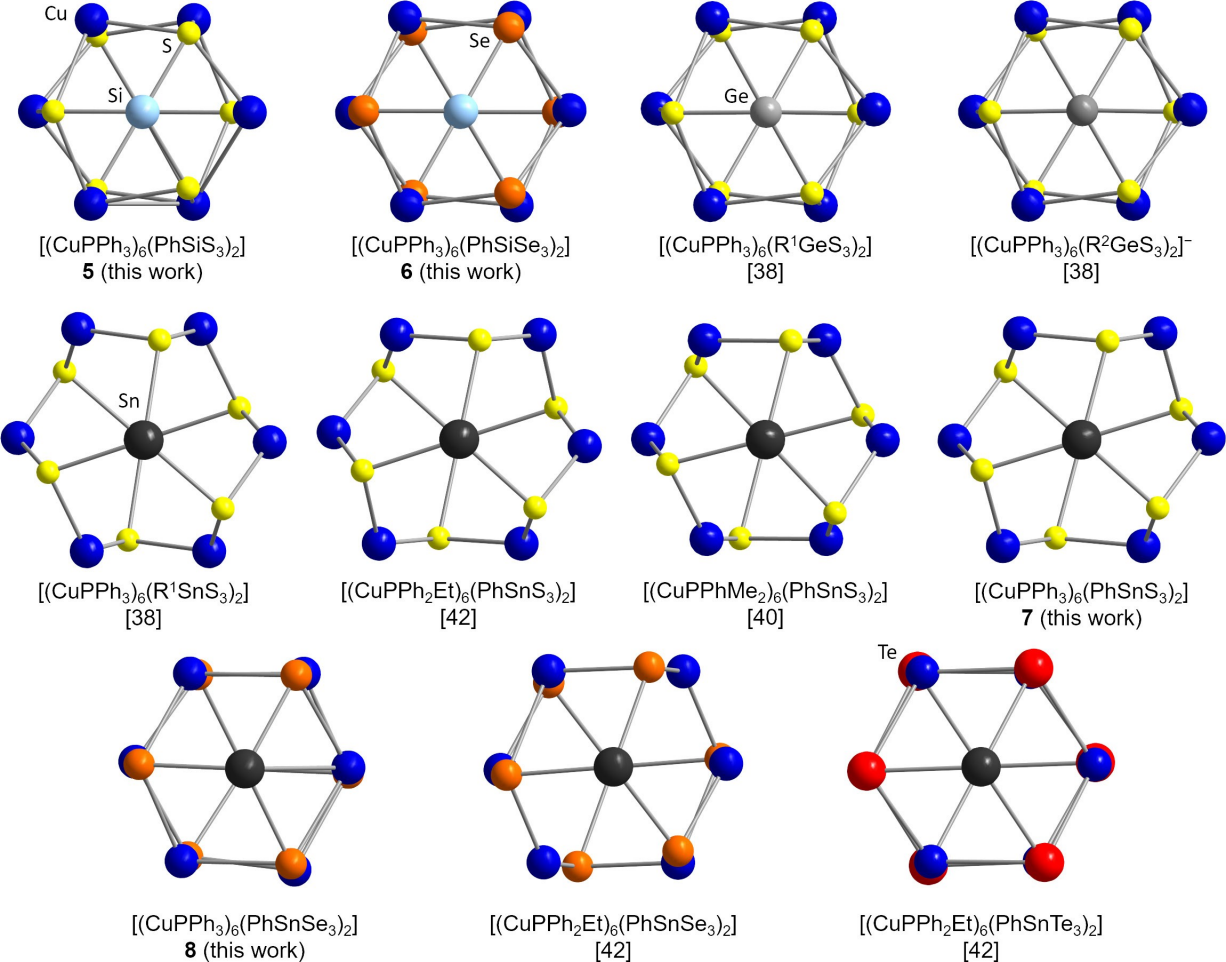
Comparison of the inorganic cluster cores of all known clusters [(CuPPh_3‐x_R′_x_)_6_(RTE_3_)_2_], viewed along the T⋅⋅⋅T axis. R^1^=CMe_2_CH_2_ C(O)Me; R^2^=C_2_H_4_COOH).

In this context, the observed differences in the symmetry of the cluster cores also caught our interest, so we compared all crystallographically determined species of the general types [(CuPPh_2_R′)_6_(RTE_3_)_2_] or [(CuPPhR′_2_)_6_(RTE_3_)_2_] (with the exception of [(CuPPh_3_)_6_(R^Fc^SnS_3‐x_Se_x_)_2_] (R^Fc^=ferrocenyl),^[49]^ which exhibits a high degree of distortion due to the statistical disorder of S and Se atoms in the cluster core). The inorganic cluster cores are shown in Figure 4 (viewed along the T⋅⋅⋅T axis). In this projection, one recognizes two hexagons, Cu_6_ and E_6_, with different degrees of distortion and relative orientations to each other. This way, two groups of conformers can be defined. In one of them, the two hexagons are oriented in an ecliptic manner (combinations Ge/S, Si/S, Si/Se and Sn/Te), while in the other one, they are tilted against each other (Sn/S) by nearly (ideal) 30°.

For the elemental composition Sn/Se, neither of the two classifications fit perfectly, due to distortions within the two hexagons. The major impact on the cluster core conformations seems to be based on the ratios of the covalent radii^[63]^ of the elements r_T_/r_E_, with the smallest quotients (hence more similar radii) of Sn/Te (1.01), Ge/S (1.14), Si/S (1.06), and Si/Se (0.93) leading to highest‐symmetry structures, the largest quotient (hence, most different radii) of Sn/S (1.32) causing a twisted conformation, and the elemental combination of Sn/Se (1.16) falling in between. To further examine this, we performed density functional theory (DFT) calculations with the program system Turbomole.[^64^, ^65^, ^66^, ^67^, ^68^, ^69^, ^70^, ^71^, ^72^, ^73^] We optimized structures of clusters with either Ph or Me substituents attached to the inorganic core (see Figure S33). The results confirmed the assumptions above. The only exceptions were found for [(CuPMe_3_)_6_(MeTS_3_)_2_] (T=Si, Ge), where the tilted conformation was favored. This implied that very small substituents play at least a minor role, which conformer is preferred.

**Figure 5:**
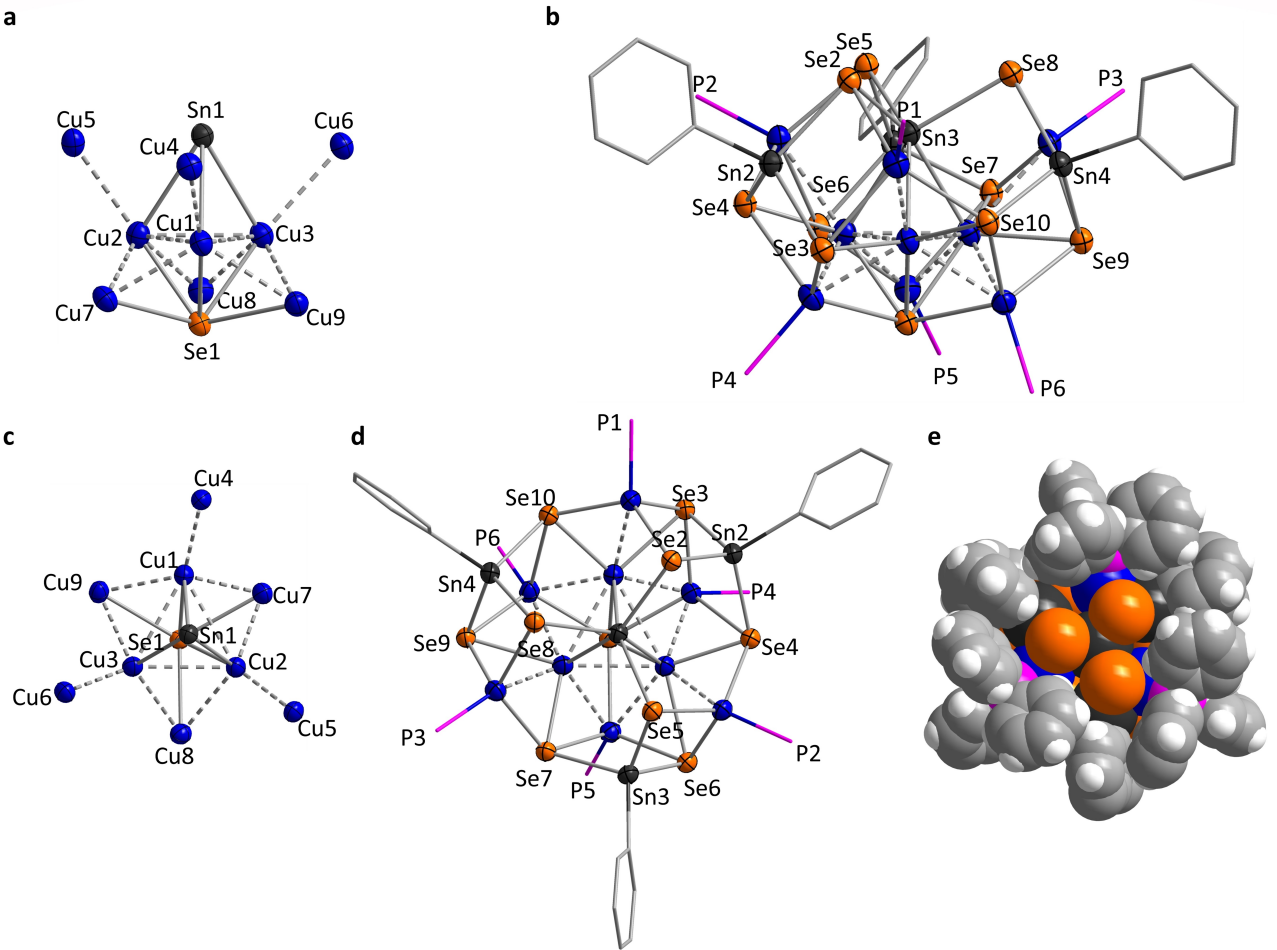
Molecular structure of **9**, illustrated by depicting the {Cu_9_SnSe} cluster core (a) and the whole molecule (b) in a view perpendicular to the Sn1⋅⋅⋅Se1 axis, the cluster core (c), the whole molecule (d), and a space‐filling model (e) viewed approximately along the Sn1⋅⋅⋅Se1 axis. Displacement ellipsoids are shown at 50 % probability, H atoms and phenyl groups of the PPh_3_ ligands are omitted for clarity. P and C atoms are drawn as wires. Note that the atom labels given in a) and c) are omitted in b) and d) for clarity. Relevant structural parameters are mentioned in the text.

A reaction of the Sn/Se‐based precursor **4** with [Cu(PPh_3_)Cl] including a layering step with *n*‐hexane affords orange‐red crystals of compound **9** ⋅ 1.65 DCM (monoclinic space group *P*2_1_/*c*). The cluster in this compound possesses idealized *C*_3v_ symmetry in its inorganic core that embeds a nearly equilateral Cu_3_ triangle (Cu^A^=Cu1, Cu2, Cu3; Cu⋅⋅⋅Cu 2.7598(8)–2.7961(8) Å; Figure 5). The Cu^A^ triangle is capped on one side by a Sn atom (Sn1), which lacks the organic ligand and is thus a formal Sn^II^ atom. We suppose that a redox process took place during the reaction of Na_3_[SnSe_3_Ph] with [Cu(PPh_3_)_3_Cl], thereby affording PhCl and Sn^II^. While the spectroscopic data did not allow the identification of corresponding side products in the present case, they were observed previously in similar reactions,[^35^, ^37^, ^74^] which we take as a strong indication for this process to regularly occur in this system. Overall, Sn1 is situated in a (distorted) octahedral environment of three Se and three Cu atoms. The other side of the Cu_3_ triangle is capped by a “naked” bridging Se ligand (Se1) that also resulted from the partial degradation of the [PhSnSe_3_]^3−^ anions during the reaction. Together, the five atoms adopt the shape of a bi‐pyramidal {Cu_3_SnSe} subunit. Two further fairly regular triangles of Cu atoms, yet with larger Cu⋅⋅⋅Cu distances are situated on either sides of the central triangle: one Sn1 (Cu^B^=Cu4, Cu5, Cu6; Cu^B^⋅⋅⋅Cu^B^ 6.018(1)–6.189(1) Å; Cu^A^⋅⋅⋅Cu^B^ 2.7203(9)–2.7801(7) Å) and on the Se1 side of the central triangle (Cu^C^=Cu7, Cu8, Cu9; Cu^C^⋅⋅⋅Cu^C^ 4.267(1)–4.294(1) Å; Cu^A^⋅⋅⋅Cu^C^ 2.5420(8)–2.5922(8) Å), The resulting {Cu_9_SnSe} subunit (with Se1 being μ^6^‐bridging in the sum) is illustrated in Figures 5a and 5c. While the Cu^A^ atoms released their phosphine ligands during the reaction, the Cu^B^ and Cu^C^ atoms bind one PPh_3_ group each (involving P1–P6).

The structural parameters of the three {PhSnSe_3_} units in compound **9** (involving Sn2−Sn4) do not deviate much from those in **4** (Sn−Se: 2.4979(8)–2.5560(7) Å; Se−Sn−Se 102.81(2)–112.64°). They coordinate in the following way to the copper atoms and Sn1 of the {Cu_9_SnSe} subunit: One of the Se atoms of each group (Se2, Se5, Se8) binds to Sn1 and two of the Cu^B^ atoms, thus acting as μ^3^‐bridging ligands. The two other Se atoms per group (Se3, Se6, Se9 and Se4, Se7, Se10) coordinate in a μ^4^‐type fashion to Sn1 and each one of the Cu^A^, Cu^B^, and Cu^C^ atoms. As can be seen in Figure 5d, these two groups of 3 Se atoms behave nearly equivalently regarding the vertical mirror planes of the idealized *C*_3v_‐symmetric cluster. However, the side view shown in Figure 5b indicates that they are actually situated on slightly different “heights” of the cluster along the idealized *C*_3_ axis. The PPh_3_ groups attached to the Cu^B^ and Cu^C^ atoms serve to shield and thus kinetically stabilize the cluster core, as illustrated by the space filling model of **8** (Figure 5e). However, this representation also indicates that Se2, Se5, and Se8 are not completely buried by Ph groups, but form the bottom of a bowl‐shaped opening.

A reason for the formation of the new cluster archetype in **9** – which was obtained in two different types of solvates and apparently exclusively for the elemental combination Sn/Se – might be found in the bond dissociation energies of the T−E bonds, where Sn−Se (401 kJ/mol) ranks as the least stable bond (Si−S: 617 kJ/mol, Si−Se: 538 kJ/mol, Sn−S:467 kJ/mol).^[75]^ This, might facilitate further fragmentation/re‐organization of **8** at longer reaction times prior to crystallization of the final product.

**Figure 6:**
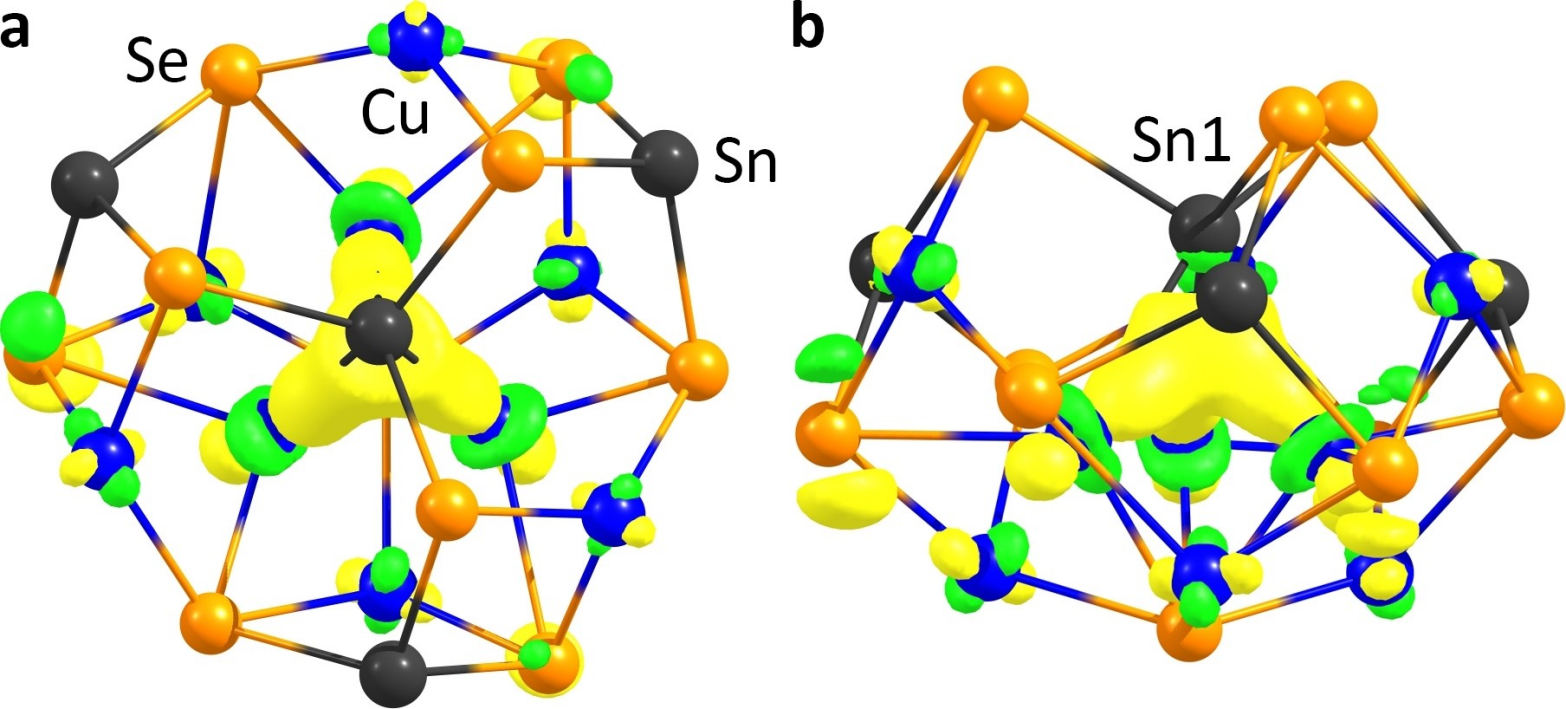
Close‐up of the LMO of the multi‐center interaction between Sn1 and the Cu^A^ atoms, in a) top‐view, b) side‐view. Contours are plotted at ±0.05 a.u.

To elucidate the character of the metal‐metal contacts within the cluster molecule, we carried out DFT calculations. Both shared electron numbers (SEN, see Table S12), calculated by means of Paboon,^[76]^ and localized molecular orbitals (LMOs), generated by means of the Boys method,^[77]^ confirm the absence of notable cuprophilic interactions. The only significant metal‐metal interactions are multi‐center interactions found between the Cu^A^ atoms and Sn1. The corresponding localized molecular orbital (LMO) is illustrated in Figure 6. The main contributions in this LMO come from the p‐type atomic orbitals of Sn1 and from the 
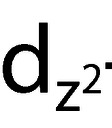
‐type atomic orbitals of the Cu^A^ atoms. As with most cluster components, the electrons generally show a large degree of delocalization over the whole inorganic core.

### Optical absorption properties

**Figure 7:**
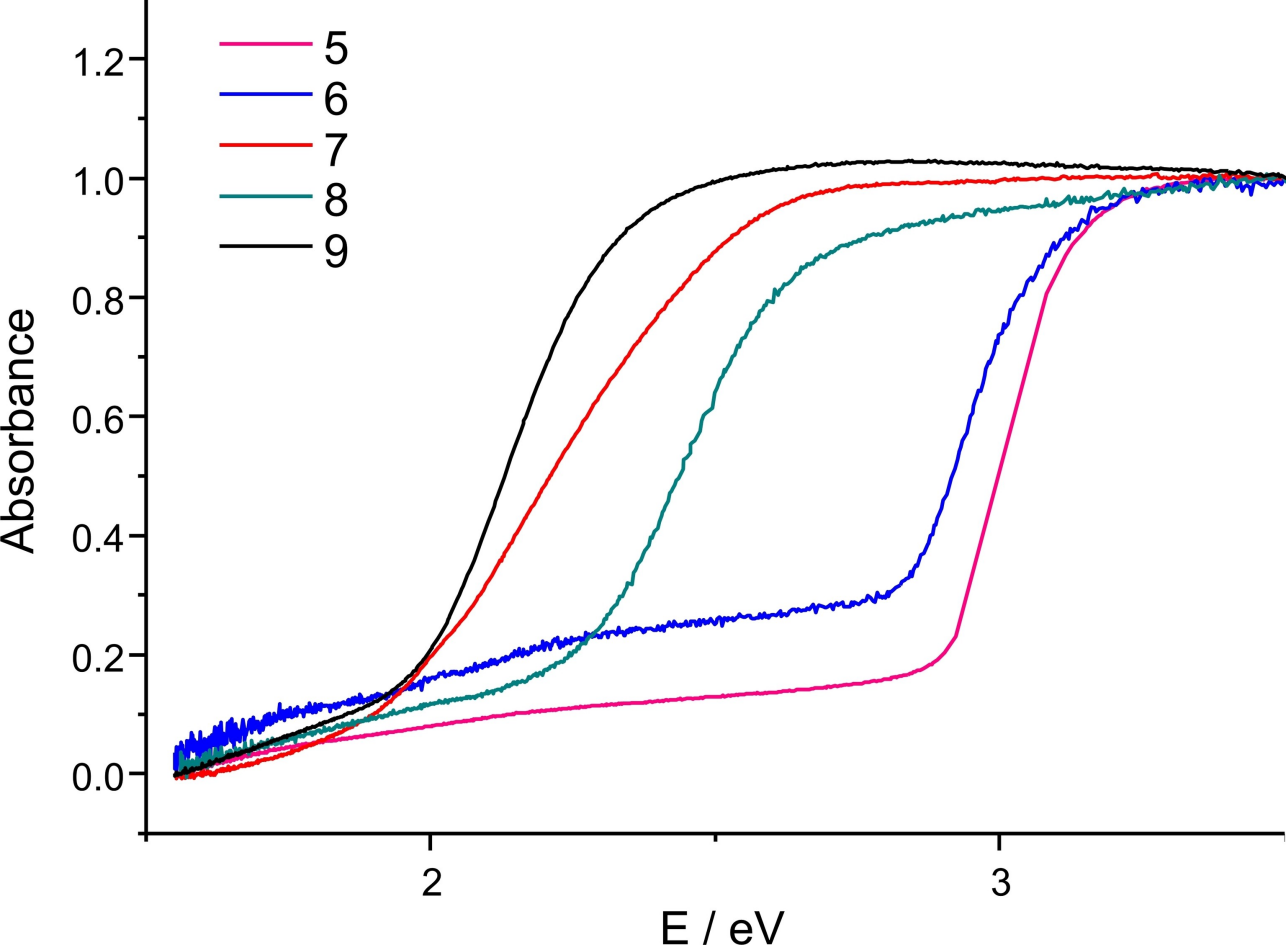
UV‐visible spectrum of pulverized single crystals of **5** (magenta), **6** (blue), **7** (red), **8** (teal), and **9** (black).

While all previously reported cluster compounds of this type exhibited a yellow to red color, including compounds **7** (orange), **8** (yellow) and **9** (orange‐red), the two unprecedented Si congeners, **5** and **6**, are colorless. To quantify this finding, we recorded UV‐vis spectra of pulverized crystalline compounds (Figure 7). As expected from the visible color, the absorption edges of **5** and **6** were found to be 2.92 eV (425 nm) and 2.86 eV (434 nm), respectively. The absorption lines reach saturation at around 3 eV, in agreement with the compounds’ colorless appearance. Compounds **7** and **8** show smaller optical excitation energies, namely 2.05 eV (605 nm) and 2.33 eV (532 nm), respectively. The band gap of **9**, as that of **7**, was also observed to be 2.05 eV (605 nm). All numbers were estimated by extrapolations in the Tauc plots (Figures S28–S32).

Notably, while the band gaps are the same, the general absorption behavior of these compounds is different, which explains the slightly different colors. Our experimental findings are in agreement with the colors and optical gaps reported for related compounds [(CuPPh_2_Et)_6_(PhSnE_3_)_2_] (2.83 eV/438 nm for E=S; 2.32 eV/534 nm for E=Se; 1.71 eV/725 nm for E=Te),^[42]^ except the value for the Se compound measured by us being larger than its S congener and the reported Sn/Se cluster. Yet, it is in agreement with the macroscopic color of the compounds (see above). We ascribe this finding mainly to a significantly denser packing of the molecules in the crystal, which is obvious from a comparison of the unit cell volumes: 2853.6(3) Å^3^ for one formula unit of **7** versus 13081.6(2) Å^3^ for 4 formula units of **8**, which is equivalent to 3270.4 Å^3^ per formula unit. The unit cell volume of compound **8** thus is by ∼15 % larger per formula unit than that of compound **7**. On one hand, this equals the difference of the atomic sizes of Se versus S, but given that the chalcogen atoms represent only 30 at % of the heavy atoms, and only 4.2 at % of the non‐hydrogen atoms, we suggest that the volumes are indicative for a denser packing.

To gain more insight in the optical excitation properties, we inspected the frontier orbitals (HOMO, LUMO; Figure S34) and analyzed the excitation event by means of time‐dependent DFT calculations (TD‐DFT).[^78^, ^79^, ^80^, ^81^] Both the frontier orbitals and the non‐relaxed electronic difference densities (Figure S35) suggest that the absorption of the Si/S, Si/Se, and Sn/S clusters **5**, **6**, and **7** mainly involve transitions from Cu(3d,4s) and S(3p) or Se(4p) orbitals within the cluster core to the Ph groups of the PPh_3_ ligands. For the Sn/Se‐based cluster **8**, however, the absorption is based mainly on a Se(4p)/Cu(4a)→Sn(5s) electronic transition. Similar observations are made for the calculations of **9**, hence indicating that the Sn/Se combination of cluster atoms is fundamentally different from the other elemental combinations. Calculated molecular excitation energies amount to 2.99 eV (**5**), 2.91 eV (**6**), 3.05 eV (**7**), 2.83 eV (**8**), and 2.45 eV (**9**), respectively (see Table 1 and spectra shown in Figure S36).

**Table 1:** Lowest excitation energies as observed experimentally, Δ*E*_exp_, or by means of TD‐DFT calculations, Δ*E*_TD‐DFT_, and HOMO‐LUMO gap energies, Δ*E*_HOMO‐LUMO_, obtained from these calculations. All values are given in eV.

Compound	Δ*E*_exp_	Δ*E*_TD‐DFT_	Δ*E*_HOMO‐LUMO_
**5**	2.92	2.99	3.59
**6**	2.86	2.92	3.51
**7**	2.05	3.05	3.69
**8**	2.33	2.83	3.66
**9**	2.05	2.45	3.24

Although the lowest absorption energies of **5** and **6** are perfectly reproduced by the calculations, we find a systematical overestimation of the values for the organotin‐based clusters **7**, **8**, and **9**. The deviation towards slightly larger energies amounts to 0.4 (**9**), 0.5 (**8**) or 1.0 eV (**7**), which for the two first (and in part for the latter) case is based on the relatively complicated mixture of transitions involved in this first excitation in case of the organotin clusters. Inspection of the calculated HOMO‐LUMO gaps confirm that the results of the TD‐DFT treatment are reasonable. The large deviation observed for compound **7**, however, is in agreement with the finding that the experimental excitation energy is exceptionally small in comparison with the homologous compounds (see above). While we cannot explain this fact with certainty to date, we can exclude that it is due to any impurities, as the pure single crystals indeed possess an exceptionally intense color. The best explanation we can offer at the moment, is a thus the density of the packing of molecules in the crystal (see above), which is not considered in the DFT calculations of isolated molecules. Our assumption is supported by the fact that as‐prepared crystals of the two solvates of compound **9** also differ in their color (see Figures S16 and S18), as a consequence of different amounts of solvent and correspondingly different crystal structures. Upon pulverization and evaporation, the colors become the same (orange‐red), in agreement with the optical absorption behavior shown in Figure 7.

## Conclusion

Reactions of Na_3_[PhTE_3_] (T=Si, Sn; E=S, Se) – formed from [(PhT)_4_E_6_] and Na_2_E – with [Cu(PPh_3_)_3_Cl] were successful for all four elemental combinations of T and E, yet with two kinds of products. Clusters of the type [(CuPPh_2_R′)_6_(RTE_3_)_2_] (R′=R here) with a known topology were obtained for all elemental compositions. The structures differ in detail, which was attributed to the ratio of the covalent radii of T and E according to a quantum chemical study on the whole series of related clusters. The fourth T/E combination (Sn/Se) additionally afforded a new cluster archetype, [(CuPPh_3_)_6_(PhSnSe_3_)_3_Cu_3_SnSe], with idealized *C*_3v_ symmetry. The larger cluster most probably formed upon partial degradation of the [PhSnSe_3_]^3−^ precursor unit. Quantum chemical studies indicated a high degree of electron delocalization in the inorganic cluster core, including Cu−Sn bonding, yet no significant bonding interactions between the Cu atoms. Notably, and in contrast to the clusters comprising Sn atoms, all Si congeners are found to be colorless compounds, which was quantified by measurements of solid state UV‐visible absorption spectra. Time‐dependent density functional theory calculations helped to analyze the electronic absorption properties and identify (a) fundamental differences between the Sn/Se‐based clusters and all others, and (b) clear indications for a difference of the molecular and the solid state systems – reflected by corresponding findings in the experimental spectra. While this study was dedicated to studying formation and geometric as well as electronic structures in detail, future studies aim at an investigation of the material properties of the compounds, which includes thermal properties measurements and the potential of forming ternary Cu/T/E phases.

## Experimental Section

All details of the syntheses, characterization and quantum chemical studies of the compounds discussed in this work are provided in the Supporting Information.

## Conflict of interest

The authors declare no conflict of interest.

## Supporting information

As a service to our authors and readers, this journal provides supporting information supplied by the authors. Such materials are peer reviewed and may be re‐organized for online delivery, but are not copy‐edited or typeset. Technical support issues arising from supporting information (other than missing files) should be addressed to the authors.

Supporting Information
