# Supplementary material for: Systematic Access of Ternary Organotetrel‐Copper Chalcogenide Clusters by [PhTE3]3− Anions (T=Si, Sn; E=S, Se)
Source: Chemistry. 2021 Jun 22;27(43):11167–74. doi: 10.1002/chem.202101139 (PMC8453927; doi:10.1002/chem.202101139)
Supplement: Supplementary file 1 — Supporting Information [file CHEM-27-11167-s001.pdf]

Chemistry–A European Journal

Supporting Information

Systematic Access of Ternary Organotetrel-Copper Chalcogenide Clusters by $[PhTE_3]^{3-}$ Anions (T = Si, Sn; E = S, Se)

Niklas Rinn, Lukas Guggolz, Han Yu Hou, and Stefanie Dehnen*

Synthesis details

General:

All synthesis steps were carried out under argon atmosphere and exclusion of external moisture. [$(PhSi)_4S_6$], [$(PhSn)_4S_6$], [$(PhSn)_4Se_6$], [$Cu(PPh_3)_3Cl$], and **A** were prepared according to procedures reported in the literature.[1–4].

Synthesis of [$(PhSi)_4Se_6$] (1)

A solution of $PhSiCl_3$ (0,95 mL, 5.97 mmol) in 10 mL of THF was added to a suspension of Na_2Se (1.12 g, 8.99 mmol) in 10 mL of THF at 0 °C and stirred for 18 h, which resulted in the formation of a slightly yellow solution. The solvent was removed *in vacuo*, and the product was extracted by toluene. The pure product could be obtained from this solution by removal of the solvent (1.08 g, yield: 81%). By re-dissolving in THF and layering with *n*-hexane in a 1:1 ratio, crystals of **1** were obtained after 3 days.

^{1}H NMR (300 MHz, $CDCl_3$, 25 °C) δ = 7.55 (m, 3H, *Ph para, ortho*) 7.94 (m, 2H, *Ph meta*) ppm; ^{13}C NMR (75 MHz, $CDCl_3$, 25 °C) δ = 128.98 (*ortho*), 132.33 (*para*), 133.17(*meta*), 134.81 (*ipso*) ppm; ^{29}Si NMR (60 MHz, $CDCl_3$, 25 °C) δ = −6.29 ($^{1}J_{77Se}$ = 160 Hz) ppm; ^{77}Se NMR (95 MHz, $CDCl_3$, 25 °C) δ = −92.28 ($^{1}J_{29Si}$ = 160 Hz).

Synthesis of $Na_3[PhSiS_3]$ (2)

305 mg (0.50 mmol) of [$(PhSi)_4S_6$] and 232.7 mg (2.98 mmol) of Na_2S were suspend in 20 mL of acetonitrile, and the mixture was stirred at room temperature for 24 h. The solvent was subsequently removed *in vacuo* to yield the crude product that was used as received.

^{1}H NMR (300 MHz, DMSO-d6, 25 °C) δ = 6.88-7.10 (*Ph, para, ortho*) 7.94 (m, 1.6 H, *Ph meta*), 8.07 (m, 2H, *Ph meta*) ppm; ^{13}C NMR (126 MHz, DMSO-d6, 25 °C) δ = 125.20, 125,75, 134,26 ppm; ^{29}Si NMR (99 MHz, DMSO-d6, 25 °C) δ = 8.88, 10.35 ppm.

Synthesis of $Na_3[PhSiSe_3]$ (3)

55 mg (0.061 mmol) of [$(PhSi)_4Se_6$] and 46 mg (0.369 mmol) of Na_2Se were suspend in 20 mL of acetonitrile and the mixture was stirred at room temperature for 24 h. The product formed as a colorless powder, which was isolated by filtration. Owing to the poor solubility of the compound, NMR spectra could not be obtained.

Synthesis of $Na_3[PhSnSe_3]$ (4)

256 mg (0.20 mmol) of [$(PhSn)_4Se_6$] and 153 mg (1.22 mmol) of Na_2Se were suspend in 20 mL of acetonitrile, and the mixture was stirred at room temperature for 24 h. The solvent was subsequently removed *in vacuo* to yield a slightly yellow powder. The crude product was dissolved in DMF and layered with Et_2O in a 1:1 ratio. Crystals of **4**·DMF form within 3 days.

^{1}H NMR (500 MHz, DMSO-d6, 25 °C) δ = (m, 3H, *Ph para, ortho*) (m, 2H, *Ph meta*) ppm; ^{13}C NMR (126 MHz, DMSO-d6, 25 °C) δ = (*ortho*), 132.33 (*para*), 133.17(*meta*), 134.81 (*ipso*) ppm; ^{77}Se NMR (95 MHz, DMSO-d6, 25 °C) δ = -230 ppm; ^{119}Sn NMR (187 MHz, $CDCl_3$, 25 °C) δ = −228.83 ($^{1}J_{77Se}$ = 791 Hz) ppm.

Synthesis of $[(CuPPh_3)_6(PhSiS_3)_2]$ (5)

36 mg (0.13 mmol) of $Na_3[PhSiS_3]$ and 356 mg (0.40 mmol) of $[Cu(PPh_3)_3Cl]$ were suspended in 15 mL of DCM. A light orange precipitate forms within 2 h. After stirring for 24 h, the precipitate was removed, and the light yellow solution was layered with *n*-hexane in a 1:3 ratio to form crystals of **5**·2 CH_2Cl_2 after 3 days. Alternatively, the slow evaporation of the solvent through a gas phase diffusion process into an acetonitrile reservoir resulted in crystals of **5**·2.76 CH_2Cl_2. Owing to the poor solubility of the cluster compound upon crystallization, NMR spectra could not be obtained.

Synthesis of $[(CuPPh_3)_6(PhSiSe_3)_2]$ (6)

19 mg (0.046 mmol) of $Na_3[PhSiSe_3]$ and 123 mg (0.139 mmol) of $[Cu(PPh_3)_3Cl]$ were suspended in 15 mL of DCM. Within 2 h, a brown precipitate and a colorless solution have formed. After stirring for 24 h, the precipitate was removed and the colorless solution was layered with *n*-hexane in a 1:2 ratio. Crystals of **6** form within 3 days. Owing to the poor solubility of the cluster compound upon crystallization, NMR spectra could not be obtained.

Synthesis of $[(CuPPh_3)_6(PhSnS_3)_2]$ (7)

80 mg (0.22 mmol) of $Na_3[PhSnS_3]$ and 589 mg (0.67 mmol) of $[Cu(PPh_3)_3Cl]$ were suspended in 20 mL of DCM. The solution turned from orange to a deeper red with a red precipitate after stirring for 18 h. Crystals of **7**·2 CH_2Cl_2 were obtained upon filtration and layering of the filtrate with *n*-hexane in a 1:1 ratio after 3 days. Owing to the poor solubility of the cluster compound upon crystallization, NMR spectra could not be obtained.

Synthesis of $[(CuPPh_3)_6(PhSnSe_3)_2]$ (8) or $[(CuPPh_3)_6(PhSnSe_3)_3Cu_3SnSe]$ (9)

155 mg (0.31 mmol) of $Na_3[PhSnS_3]$ and 821 mg (0.93 mmol) of $[Cu(PPh_3)_3Cl]$ were suspended in 6 mL (**8**) or 20 mL (**9**) of DCM, respectively. After stirring for 18 h, a brown precipitate formed over a deep red solution. The precipitate was removed by filtration, whereupon crystals of **8** form after 1 day in the more concentrated filtrate. In contrast, crystals of **9**·1.65 CH_2Cl_2 and **9**·3.35 CH_2Cl_2 were found to form in a 1:3 ratio within one week upon layering of the more diluted filtrate with *n*-hexane. Owing to the poor solubility of the cluster compound upon crystallization, NMR spectra could not be obtained.

Crystallography

Crystals suitable for X-ray diffraction analyses were investigated at 100K either with a STOE STADIVARI diffractometer (**A**·2 EtOH, **5**·2 DCM, **7**·2 DCM, **8**·4.40 DCM, **9**·1.65 DCM, and **9**·3.35 DCM) using $Cu_{K\alpha}$ radiation (λ = 1.54186) from an X-ray micro source with X-ray optics and a Pilatus 300K Si hybrid pixel array detector or with a STOE IPDS-2T diffractometer (**A**·EtOH·H_2O, **1**, **4**·DMF, **5**·2.76 DCM, and **6**) using $Mo_{K\alpha}$ radiation and a graphite monochromator (λ = 0.71073 Å). Upon spherical absorption correction and scaling (STOE LANA), the structure solution was performed by intrinsic phasing methods, followed by full-matrix-least-squares refinement against F2, using SHELXT15, SHELXL15, and OLEX2 software.[5–7] The as of yet unreported crystal structures of **A** were obtained by dissolving **A** in water and layering with EtOH, upon which crystals formed within a few days. Ligands and solvent molecules in all Images are drawn as wires, while other atoms are drawn with displacement ellipsoids at 50% probability. H atoms are omitted for clarity. The crystallographic data are summarized in Tables S1-S4.

Table S1. Crystallographic data and refinement results of **A** (two solvates) and **1**.

Compound	**A**·2 EtOH	**A**·EtOH·0.5 H_2O	**1**
Empirical formula	$C_{10}H_{15}Na_3O_2S_3Sn$	$C_8H_{11}Na_3O_{1.5}S_3Sn$	$C_{24}H_{20}Se_6Si_4$
Fw / g·mol^{-1}	451.06	415.01	894.52
Crystal color and shape	colorless prism	colorless block	colorless block
Crystal size / mm^3	0.33×0.31×0.17	0.13×0.09×0.08	0.18×0.13×0.11
Crystal system	monoclinic	monoclinic	Monoclinic
Space group	$P2_1/c$	$P2_1/n$	$P2_1/c$
a / Å	16.3431(3)	7.2782(4)	13.7740(1)
b / Å	8.1580(2)	33.630(3)	12.2610(1)
c / Å	13.4800(3)	12.0857(7)	17.5438(1)
	90	90	90
β / °	109.062(2)	92.662(5)	96.767(1)
γ / °	90	90	90
V / Å^3	1698.70(7)	2955.0(3)	2942.21(4)
Z	4	8	4
ρ_{calcd} / g·cm^{-3}	1.764	1.866	2.019
μ / mm^{-1}	(Cu Kα) 16.117	(Mo Kα) 2.220	(Cu Kα) 12.340
Absorption correction type	sphere	sphere	sphere
min. / max. transmission	0.077 / 0.273	0.2994 / 0.6756	0.062 /0.271
2θ range / deg	5.60 / 60.08	3.584 / 53.624	5.08 / 150.89
no. of meas. reflns.	27171	20000	100294
R(int)	0.0606	0.1564	0.0268
Indep. reflns.	3434	6249	6019
Indep. reflns. ($I > 2\sigma(I)$)	2970	2867	5663
no. of parameters	174	309	307
$R1$ ($I > 2\sigma(I)$) / w$R2$ (all data)	0.0398 / 0.1085	0.0618 / 0.1497	0.0207 / 0.0476
S (all data)	0.989	0.867	1.115
Max. peak / hole / e^{-}· Å^3	1.414 / –0.764	1.413 / –1.599	0.37 / –0.70
CCDC number	2033634	2033635	2033637

Table S2. Crystallographic data and refinement results of **4** and **5** (two solvates).

Compound	**4**·DMF	**5**·2 CH_2Cl_2	**5**·2.76 CH_2Cl_2
Empirical formula	$C_9H_{11}NNa_3OSe_3Sn$	$C_{122}H_{104}Cl_4Cu_6P_6S_6Si_2$	$C_{122.76}H_{105.53}Cl_{5.53}Cu_6P_6S_6Si_2$
Fw / g·mol^{-1}	848.87(9)	2527.45	2592.41
Crystal color and shape	Clear colorless plate	colorless prism	colorless block
Crystal size / mm^3	0.32×0.17×0.09	0.20×0.19×0.18	0.30x0.25x0.22
Crystal system	triclinic	monoclinic	triclinic
Space group	$P\bar{1}$	$P2_1/c$	$P\bar{1}$
a / Å	7.8026(5)	13.5379(2)	17.0385(5)
b / Å	7.8343(5)	27.0419(2)	17.1157(5)
c / Å	15.4487(9)	16.5616(2)	25.1201(11)
α / °	94.102(5)	90	97.559(3)
β / °	94.178(5)	109.735(1)	103.54(3)
γ / °	114.892(4)	90	115.007(2)
V / Å^3	848.87(9)	5706.9(1)	6249.9(4)
Z	2	2	2
ρ_{calcd} / g·cm^{-3}	2.245	1.471	1.378
μ / mm^{-1}	(Mo Kα) 7.999	(Cu Kα) 4.481	(Mo Kα) 1.363
Absorption correction type	sphere	sphere	sphere
min. / max. transmission	1.000 / 1.000	0.0705 /0.2328	0.2649 / 0.6281
2θ range / deg	2.66 / 54.52	3.268 / 75.065	3.72 / 54.28
no. of meas. reflns.	12731	102341	18127
R(int)	0.0442	0.0372	0.0684
Indep. reflns.	3592	11408	6253
Indep. reflns. ($I > 2\sigma(I)$)	3242	9819	2871
no. of parameters	165	658	1343
*R*1 ($I > 2\sigma(I)$) / w*R*2 (all data)	0.0335 / 0.0911	0.0302 / 0.0862	0.0515 / 0.1432
S (all data)	1.044	1.066	0.9590
Max. peak / hole / e$^-$· Å^3	2.613 / –1.204	0.920 / –0.937	1.121 / –0.801
CCDC number	2033636	2033737	2033738

Table S3. Crystallographic data and refinement results of **6**, **7** and **8**.

Compound	**6**	**7**·2 CH_2Cl_2	**8**·4.40 CH_2Cl_2
Empirical formula	$C_{120}H_{10}Cu_6P_6Se_6Si_2$	$C_{122}H_{104}Cl_4Cu_6P_6S_6Sn_2$	$C_{124.4}H_{106.8}Cl_{8.8}Cu_6P_6Se_6Sn_2$
Fw / g·mol^{-1}	2638.99	2708.65	2708.65
Crystal color and shape	Colorless block	orange block	yellow block
Crystal size / mm^3	0.18×0.10×0.08	0.29×0.22×0.20	0.39×0.19×0.14
Crystal system	triclinic	triclinic	monoclinic
Space group	$P\bar{1}$	$P\bar{1}$	$P2_1/c$
a / Å	13.9360(4)	14.0851(6)	27.8989(3)
b / Å	14.1881(4)	14.1633(8)	19.39370(10)
c / Å	15.9045(5)	16.5756(7)	27.0222(3)
α / °	65.550(2)	68.650(4)	90
β / °	68.954(2)	69.296(3)	116.5260(10)
γ / °	88.054)2)	89.473(4)	90
V / Å^3	2647.3(1)	2853.6(3)	13081.6(2)
Z	1	1	4
ρ_{calcd} / g·cm^{-3}	1.655	1.576	1.621
μ / mm^{-1}	(Mo Kα) 1.363	(Cu Kα) 7.678	(Cu Kα) 7.678
Absorption correction type	Sphere	sphere	sphere
min. / max. transmission	0.7353 / 0.7373	0.0036 / 0.2532	0.0063 / 0.0418
2θ range / deg	3.04 / 54.50	6.18 / 151.90	7.312 / 152.852
no. of meas. reflns.	26359	25830	170634
R(int)	0.0418	0.1217	0.0395
Indep. reflns.	22260	11622	26991
Indep. reflns. ($I > 2\sigma(I)$)	8821	7187	21009
no. of parameters	631	658	1397
$R1$ ($I > 2\sigma(I)$) / w$R2$ (all data)	0.0337 / 0.0886	0.1163 / 0.3191	0.0447 / 0.1279
S (all data)	0.9780	1.0680	1.094
Max. peak / hole / e^{-}· Å^3	0.980 / –1.020	1.661 / –1.586	1.764 / –1.448
CCDC number	2033739	2033731	2053513

Table S4. Crystallographic data and refinement results of **9** (two solvates).

Compound	**9**·1.65 CH_2Cl_2	**9**·3.35 CH_2Cl_2
Empirical formula	$C_{127.65}H_{108.30}Cl_3Cu_9P_6Se_{10}Sn_4$	$C_{129.35}H_{111.69}Cl_{6.69}Cu_9P_6Se_{10}Sn_4$
Fw / g·mol^{-1}	3781.14	3925.21
Crystal color and shape	orange plates	red needles
Crystal size / mm^3	0.16×0.16×0.14	0.32×0.17×0.12
Crystal system	monoclinic	triclinic
Space group	$P2_1/c$	$P\bar{1}$
a / Å	14.0689(2)	14.18950(10)
b / Å	30.3836(3)	16.42190(10)
c / Å	30.6446(3)	30.9720(2)
α / °	90	102.1620(10)
β / °	91.1610(10)	90.2050(10)
γ / °	90	100.0590(10)
V / Å^3	13096.8(3)	6940.51(9)
Z	4	2
ρ_{calcd} / g·cm^{-3}	1.918	1.878
μ / mm^{-1}	(Cu Kα) 12.340	(Cu Kα) 12.26
Absorption correction type	sphere	Sphere
min. / max. transmission	0.0065 /0.0405	0.0219 /0.0044
2θ range / deg	5.68 / 60.08	5.60 / 150.56
no. of meas. reflns.	241329	199686
R(int)	0.0884	0.0382
Indep. reflns.	26679	27855
Indep. reflns. ($I > 2\sigma(I)$)	19317	25624
no. of parameters	1451	1506
$R1$ ($I > 2\sigma(I)$) / w$R2$ (all data)	0.0379 / 0.0979	0.0382 / 0.0984
S (all data)	0.943	0.943
Max. peak / hole / e$^-$· Å^3	1.92 / –1.89	4.581 / –2.334
CCDC number	2033678	2033719

Crystal structure of A·2 EtOH

The highest peak of residual electron density on the difference Fourier map (1.414 $e^{-}/Å^{3}$) is found 0.931 Å apart from Sn1 on the bond to S1. A cutout of the crystal structure is shown in Figure S1. **A**·2 EtOH forms extended chains through Na–S (2.791(2) – 2.848(2) Å) contacts and bridging ethanol ligands along the *b* axis with its organic ligands facing in *a* direction and the chains stacking along the *c* axis. When considering the shorter Na–S inter chain contacts (2.926(2) – 2.942(2) Å), the structure can also be described as a layers along the *b* plane. A list of selected structural parameters are given in Table S5.

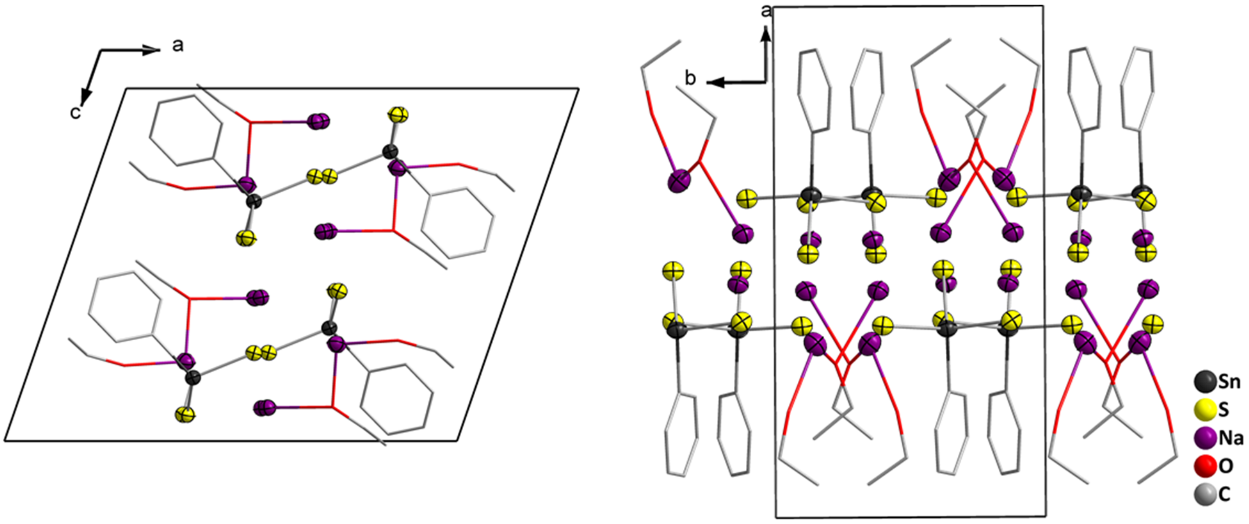

Figure S1: Cutout of the crystal structure of **A**·2 EtOH viewed along the *b* (left) and *c* axis (right).

Crystal structure of A·EtOH·0.5 H_2O

The highest peak of residual electron density on the difference Fourier map (1.414 e^{-}/Å^3) is found 1.489 Å apart from Sn1. A cutout of the crystal structure is shown in Figure S2. **A**·EtOH·0.5 H_2O forms extended layers through Na–S contacts and bridging water ligands along the *b* plane with its organic ligands facing towards the void between the layers. A list of selected structural parameters are given in Table S5.

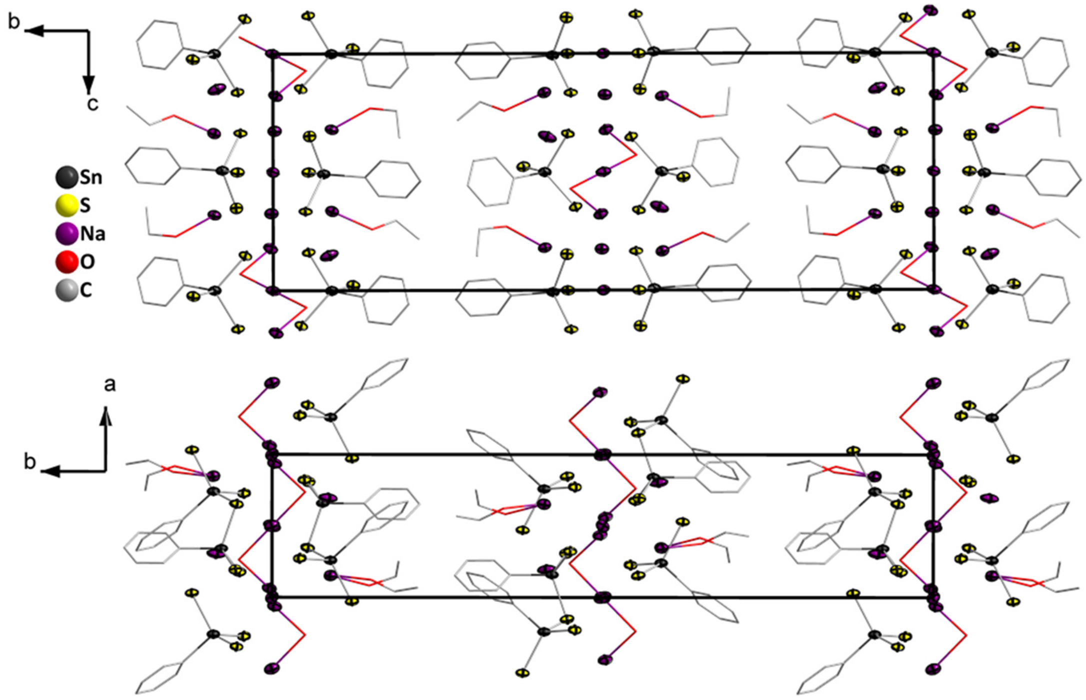

Figure S2: Cutout of the crystal structure of **A**·2 EtOH·0.5 H_2O viewed along the *a* axis (top) and the *c* axis (bottom).

Table S5. Structural parameters in **A**·2 EtOH and **A**·EtOH·0.5 H_2O

Parameter (Å / °)	**A**·2 EtOH	**A**·EtOH·0.5 H_2O
Sn1–S1	2.373(1)	2.372(2)
Sn1–S2	2.382(1)	2.363(3)
Sn1–S3	2.371(1)	2.395(3)
Sn2–S4		2.382(2)
Sn2–S5		2.392(2)
Sn2–S6		2.360(3)
Sn1–C1	2.144(4)	2.14(1)
Sn2–C7		2.13(1)
Na1–S1	2.816(2)	2.742(5)
Na1–S1^{A}	2.923(2)	
Na1–S2^{A}	2.848(2)	
Na1–S2^{B}	2.937(2)	
Na1–S3^{C}	2.942(2)	
Na1–S3		2.755(5)
Na1–S5		2.747(5)
Na2–S1	2.791(2)	
Na2–S1^{D}	2.933(2)	
Na2–S2^{E}	3.187(2)	
Na2–S2		2.804(5)
Na2–S3		2.824(5)
Na2–S3^{B}	2.926(2)	
Na2–S3^{D}	2.918(2)	
Na2–S5		3.082(5)
Na2–S6^{G}		2.883(5)
Na3–S1^{H}	3.014(2)	
Na3–S2	2.807(2)	
Na3–S3		2.733(4)
Na3–S3^{D}	2.938(2)	
Na3–S4^{I}		2.756(5)
Na3–S5		2.760(5)
Na4–S2^{F}		2.931(5)
Na4–S3^{C}		3.030(5)
Na4–S4^{J}		2.950(5)
Na4–S4		3.025(5)
Na4–S5		2.882(5)
Na4–S6^{J}		2.911(5)
Na5–S2^{K}		2.780(5)
Na5–S4^{I}		2.805(4)
Na5–S6		2.794(4)
Na6–S4^{J}		3.119(3)
Na6–S5		2.931(3)
Na6–S6		2.936(3)
Na7–S2		2.866(3)
Na7–S3		2.849(3)

A: 1−X, −1/2+Y, 2−Z; B: +X, 1/2−Y, -1/2+Z; C: 1+X, +Y, +Z; D: 1−X, 1/2+Y, 3/2−Z; E: +X, 3/2−Y, -1/2+Z; F: 1−X, 1−Y, 1−Z; G: 1−X, 1−Y, 2−Z; H: +X, 1+Y, +Z; I: −1+X, +Y, +Z; : 2-X, 1−Y, 2−Z; K:+X, +Y, 1+Z.

Crystal structure of 1

The highest peak of residual electron density on the difference Fourier map (0.37 $e^{-}/Å^{3}$) is found 0.771 Å apart from C1 near the bond to C2. An image of the crystals of **1** is shown in Figure S3, and a cutout of the crystal structure is shown in Figure S4. A list of selected structural parameters are given in Table S6.

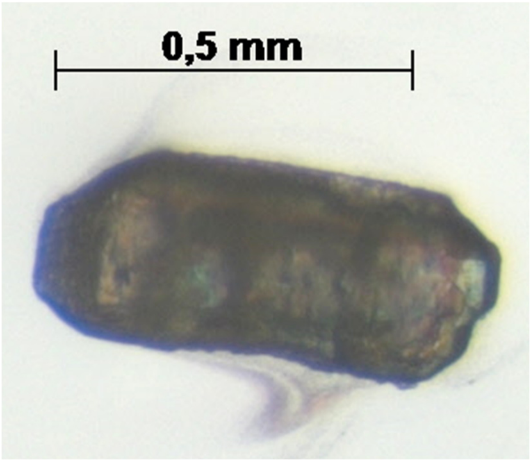

Figure S3: Light-microscopic image of a crystal of **1** (with signs of surficial decomposition).

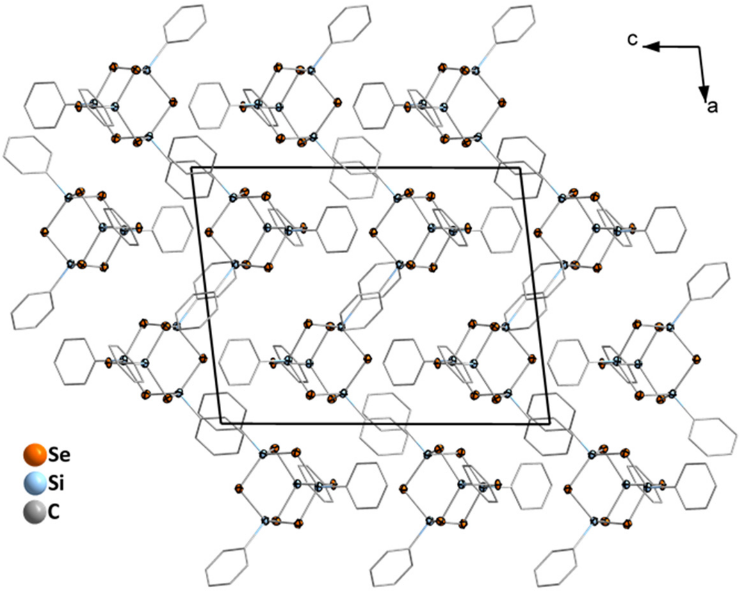

Figure S4: Cutout of the crystal structure of **1** viewed along the *b* axis.

Table S6. Structural parameters in **1**.

Parameter (Å / °)	**1**
Si1–Se1	2.2735(6)
Si1–Se2	2.2777(6)
Si1–Se3	2.2768(6)
Si2–Se1	2.2791(7)
Si2–Se4	2.2736(6)
Si2–Se6	2.2655(6)
Si3–Se2	2.2711(7)
Si3–Se4	2.2849(7)
Si3–Se5	2.2849(7)
Si4–Se3	2.2690(7)
Si4–Se5	2.2745(6)
Si4–Se6	2.2648(7)
Si1–C1	1.859(2)
Si2–C7	1.859(2)
Si3–C13	1.862(2)
Si4–C19	1.858(2)
Se1–Si1–Se2	111.80(2)
Se1–Si1–Se3	116.33(2)
Se2–Si1–Se3	111.68(2)
Se1–Si2–Se4	113.04(3)
Se1–Si2–Se6	112.21(3)
Se4–Si2–Se6	113.01(3)
Se2–Si3–Se4	113.58(3)
Se2–Si3–Se5	113.40(3)
Se4–Si3–Se5	112.86(3)
Se3–Si4–Se5	113.83(3)
Se3–Si4–Se6	111.54(3)
Se5–Si4–Se6	113.79(3)
Si1–C1–C4	178.0(1)
Si2–C7–C11	173.6(1)
Si3–C13–C16	177.6(1)
Si4–C19–C22	173.5(1)

Crystal structure of 4·DMF

The highest peak of residual electron density on the difference Fourier map (2.613 $e^{-}/Å^3$) is found 1.370 Å apart from Na3. No meaningful atom or disorder could be fitted to this site. An image of the crystals of **4**·DMF is shown in Figure S5, and a cutout of the crystal structure is shown in Figure S6. **4**·DMF forms extended layers through Na–Se contacts and bridging DMF molecules along the *b* plane with its organic ligands facing towards the void between the layers. A list of selected structural parameters are given in Table S7.

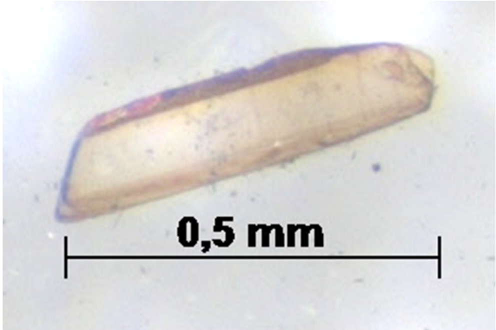

Figure S5: Light-microscopic image of a crystal of **4**·DMF.

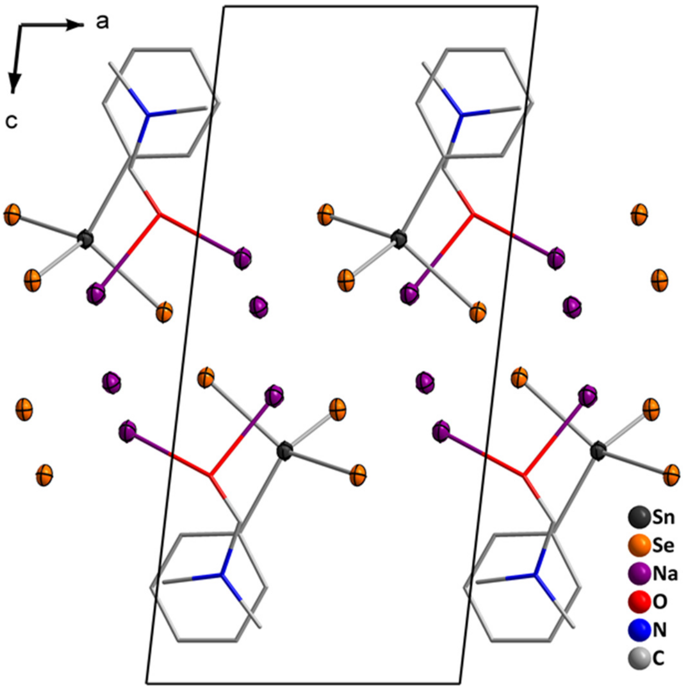

Figure S6: Cutout of the crystal structure of **4**·DMF viewed along the *b* axis.

Table S7. Structural parameters in **4**·DMF.

Parameter (Å / °)	**4**·DMF
Sn1–Se1	2.5031(5)
Sn1–Se2	2.4876(4)
Sn1–Se3	2.5270(5)
Sn1–C1	2.169(3)
Na1–Se1	2.908(1)
Na1–Se2	2.992(2)
Na1–Se3^{A}	3.083(2)
Na1–Se3^{B}	3.124(2)
Na2–Se1^{A}	2.954(2)
Na2–Se1^{C}	3.387(2)
Na2–Se2	2.923(2)
Na2–Se3^{D}	2.986(2)
Na2–Se3^{A}	2.976(1)
Na3–Se1^{E}	2.935(1)
Na3–Se1^{F}	3.091(2)
Na3–Se2^{G}	3.014(2)
Na3–Se3^{G}	3.065(1)
Na3–Se3^{A}	3.251(2)
Na1–O1	2.243(3)
Na3–O1	2.340(3)
Se1–Sn1–Se2	109.66(2)
Se1–Sn1–Se3	107.87(2)
Se2–Sn1–Se3	114.83(2)

A: 1−X, 1−Y, 1−Z; B: −1+X, +Y, +Z; C: +X, −1+Y, +Z; D: 1−X, 1−Y, +Z; E: −1+X, −1+Y, +Z; F: −X, 1−Y, 1−Z; G: −1+X, +Y, +Z.

Crystal structure of 5·2 CH_2Cl_2

The highest peak of residual electron density on the difference Fourier map (0.920 $e^{-}/Å^3$) is found 1.361 Å apart from Cl2. The solvent molecules were found to form lines in *c* direction with further electron density between the modelled sites that could not be refined to further molecules. Thus, a solvent mask in OLEX 2 was used to remove this electron density of 5.9 electrons in a void of 57.7 $Å^3$ per asymmetric unit. This would correspond to 0.25 CH_2Cl_2 per formula unit. A cutout of the crystal structure of **5**·2 CH_2Cl_2 is shown in Figure S7. The clusters can be described as balls, forming a distorted hexagonal lattice stacking along the *a* axis. A list of selected structural parameters are given in Table S8.

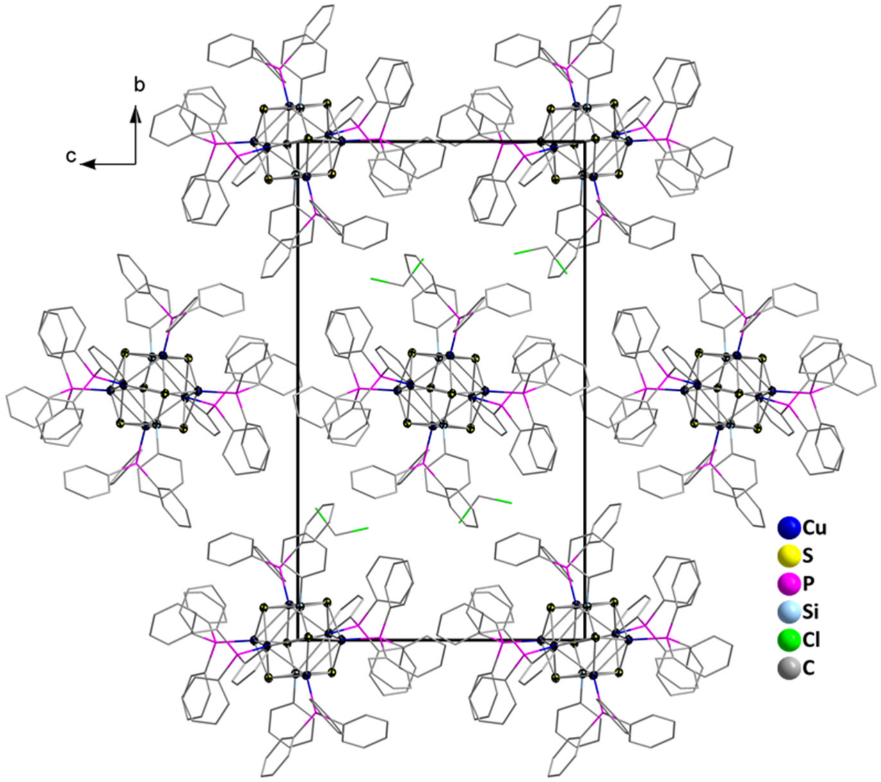

Figure S7: Cutout of the crystal structure of **5**·2 CH_2Cl_2 viewed along the *a* axis.

Crystal structure of 5·2.76 CH_2Cl_2

The highest peak of residual electron density on the difference Fourier map (1.121 $e^{-}/Å^{3}$) is found 1.165 Å apart from Cl5. A large amount of diffuse electron density was further found, that could not be modeled satisfyingly. Thus, a solvent mask in OLEX 2 was used to remove this electron count of 186.0 electrons in a void of 709.0 $Å^3$ per unit cell. This would correspond to further 1.94 CH_2Cl_2 per formula unit. The fact that these crystals lost their crystallinity extremely fast when removed from their crystallization environment and put in oil supports the presence of massive amount of volatile solvent molecules. An image of the crystals of **5**·2.76 CH_2Cl_2 is shown in Figure S8, and a cutout of the crystal structure is shown in Figure S9. The clusters can be described as balls, forming a distorted closest hexagonal packed lattice stacking along the *a* axis. A list of selected structural parameters are given in Table S8.

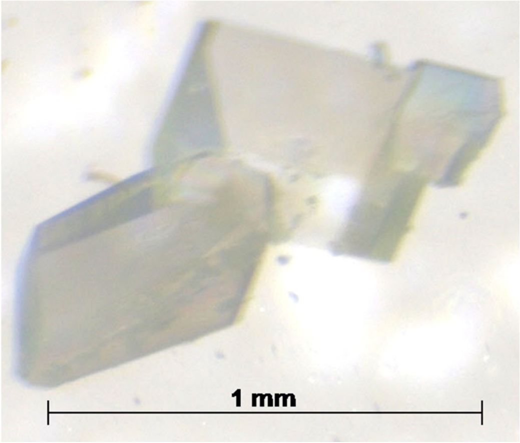

Figure S8: Image of crystals of **5**·2.76 CH_2Cl_2.

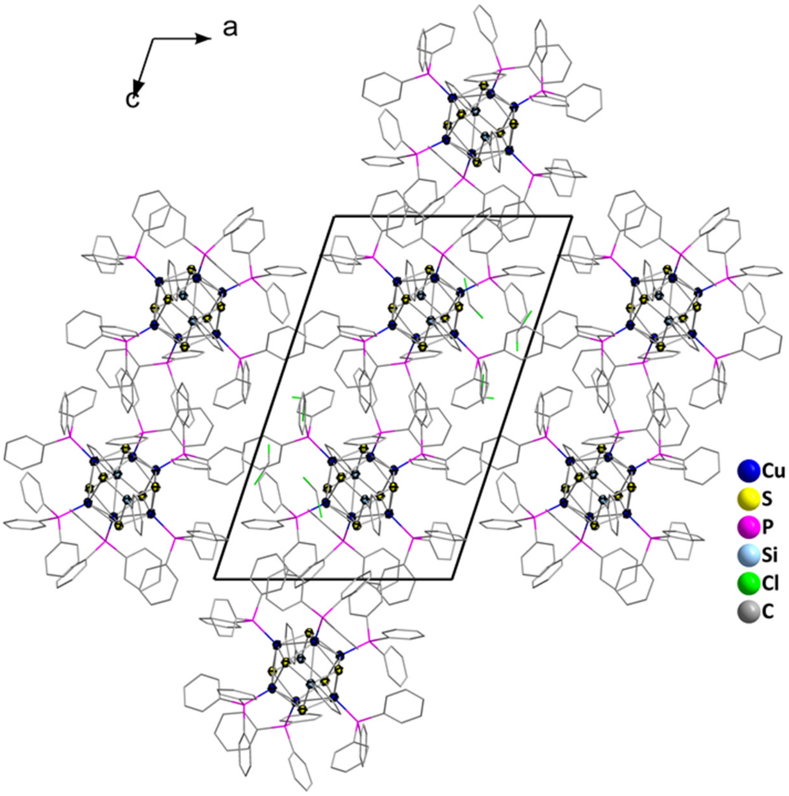

Figure S9: Cutout of the crystal structure of **5**·2.76 CH_2Cl_2 viewed along the *b* axis.

Crystal structure of 6

The highest peak of residual electron density on the difference Fourier map (0.980 $e^{-}/Å^{3}$) is found 0.908 Å apart from Cu2 close to the bond to Se2. An image of a crystals of **6** is shown in Figure S10, and a cutout of the crystal structure is shown in Figure S11. Crystal quality did degrade after several minutes when storing under oil, which is another indicator, that these crystals are most sensitive of all presented here. A list of selected structural Parameters can be seen in table S8.

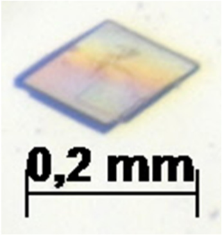

Figure S10: Image of a crystal of **6**.

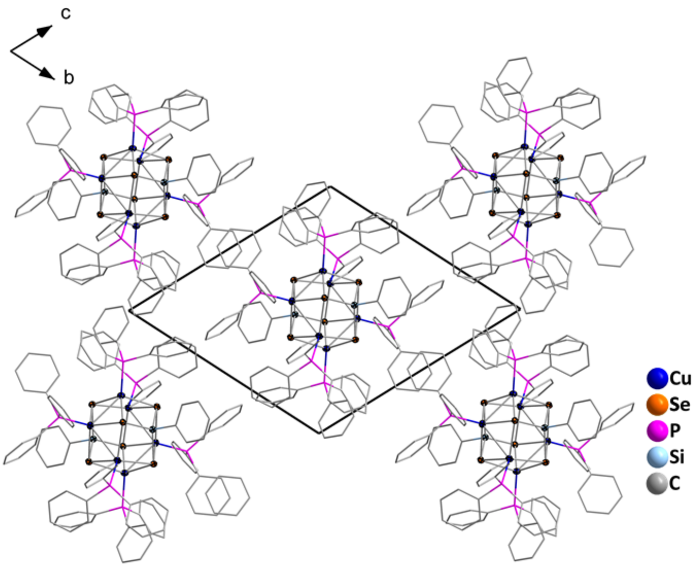

Figure S11: Cutout of the Crystal structure of **6** viewed along the *a* axis.

Crystal structure of 7·2 CH_2Cl_2

The highest peak of residual electron density on the difference Fourier map (1.661 $e^-/Å^3$) is found 0.777 Å apart from Sn1. An image of a crystals of **7** is shown in Figure S12, and a cutout of the crystal structure is shown in Figure S13. A list of selected structural parameters are given in Table S8.

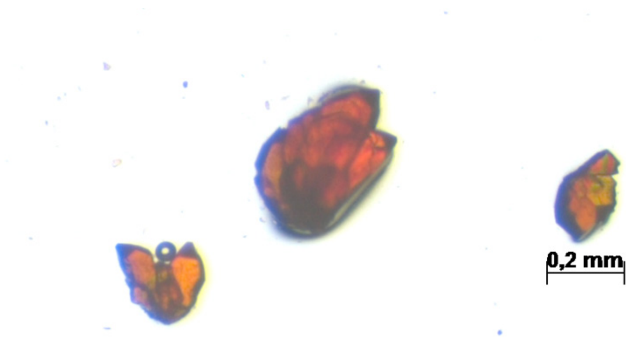

Figure S12: Image of crystals of **7**·2 CH_2Cl_2.

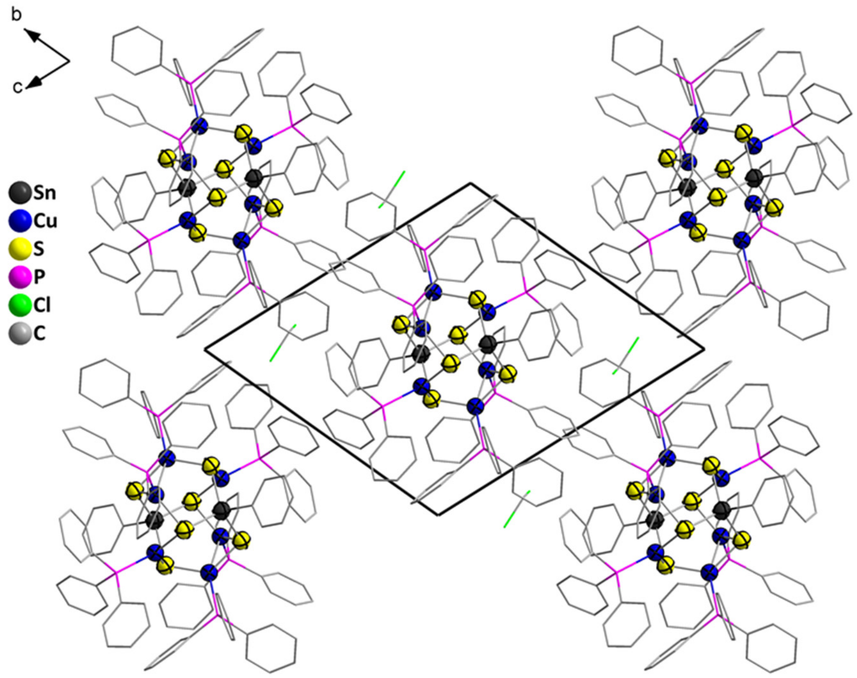

Figure S13: Cutout of the crystal structure of **7**·2 CH_2Cl_2 viewed along the *a* axis.

Crystal structure of 8·4.40 CH_2Cl_2

The highest peak of residual electron density on the difference Fourier map (1.764 $e^-/Å^3$) is found 0.696 Å apart from Cl8. A large amount of diffuse electron density was further found, that could not be modeled satisfyingly. Thus, a solvent mask in OLEX 2 was used to remove this electron count of 268.6 electrons in voids of a total 1200.4 $Å^3$ per unit cell. This would correspond to further 1.60 CH_2Cl_2 per formula unit. An image of the crystals of **8**·4.40 CH_2Cl_2 is shown in Figure S14, and a cutout of the crystal structure is shown in Figure S15. A list of selected structural parameters are given in Table S8.

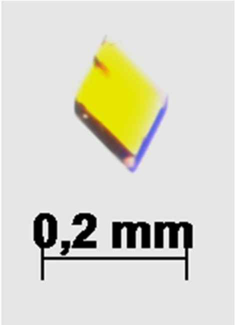

Figure S14: Image of a crystal of **8**·4.40 CH_2Cl_2.

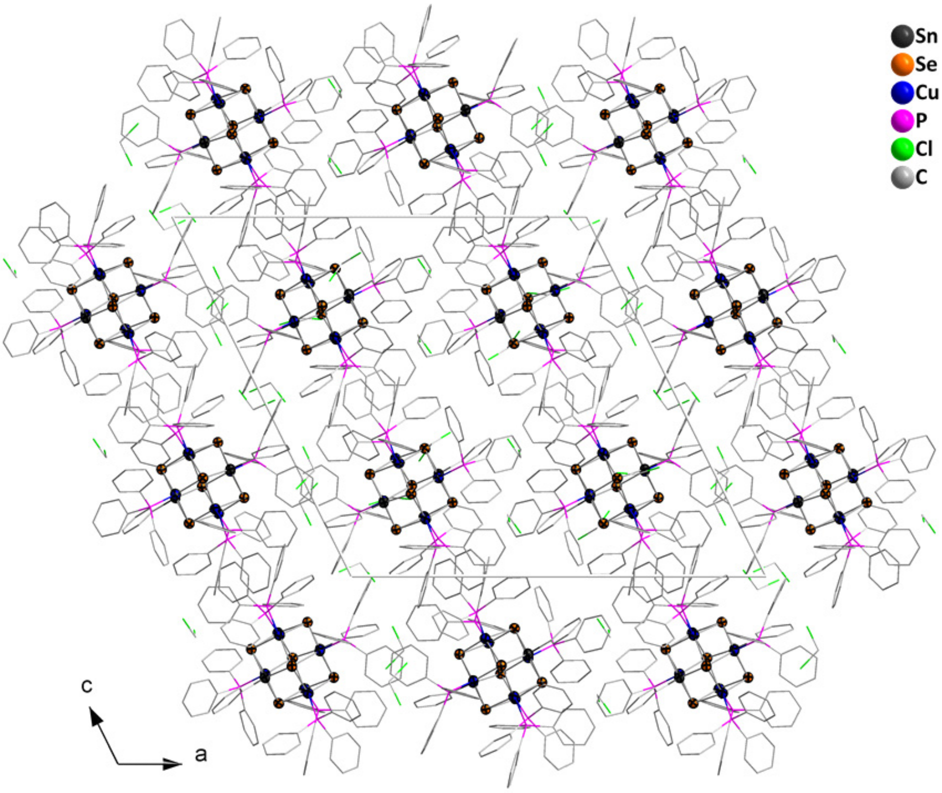

Figure S15: Cutout of the crystal structure of **8**·4.40 CH_2Cl_2 viewed along the *b* axis.

**Table S8.** Structural parameters in **5**·2 CH_2Cl_2, **5**·2.76 CH_2Cl_2, **6**, **7**·2 CH_2Cl_2, and **8**·4.40 CH_2Cl_2.					
Parameter (Å / °)	**5**·2 CH_2Cl_2	**5**·2.76 CH_2Cl_2	**6**	**7**·2 CH_2Cl_2	**8**·4.40 CH_2Cl_2
T1–E1	2.1258(8)	2.133(1)	2.2824(9)	2.395(2)	2.5142(8)
T1–E2	2.1463(8)	2.137(1)	2.2757(8)	2.407(3)	2.5180(7)
T1–E3	2.1294(6)	2.134(1)	2.2667(7)	2.403(3)	2.5178(7)
T2–E4		2.142(1)			2.5214(7)
T2–E5		2.135(1)			2.5245(8)
T2–E6		2.136(1)			2.5335(7)
T1–C1	1.883(2)	1.875(3)	1.888(4)	2.10(1)	2.132(4)
T2–C7		1.881(3)			2.134(4)
Cu1–E1	2.4345(7)	2.4917(8)	2.5344(5)	2.287(3)	2.6142(7)
Cu1–E2	2.4463(6)	2.464(1)	2.5568(5)	3.445(3)	2.6389(9)
Cu1–E4/3^{A}	2.3812(7)	2.356(1)	2.5014(7)	2.246(3)	2.4706(7)
Cu2–E2		2.3512(9)			2.4672(7)
Cu2/3^{A}–E4		2.459(1)			2.5802(9)
Cu2–E5		2.479(1)			2.6473(9)
Cu3/2–E2	2.5080(5)	2.487(1)	2.5793(5)	2.258(3)	2.6113(9)
Cu3/2–E3	2.4199(7)	2.4730(7)	2.5522(5)	3.823(3)	2.6503(7)
Cu3/2–E5/1^{A}	2.3607(7)	2.380(1)	2.4555(7)	2.255(4)	2.4481(7)
Cu4–E3		2.352(1)			2.4468(7)
Cu4–E5		2.4370(7)			2.6791(7)
Cu4–E6		2.488(1)			2.5730(9)
Cu5/3–E1	2.4960(5)	2.514(1)	2.6027(4)	3.405(4)	2.6082(9)
Cu5/3–E3	2.4591(7)	2.436(1)	2.5511(5)	2.300(3)	2.6282(9)
Cu5/3–E6/2^{A}	2.3605(7)	2.3794(9)	2.4663(7)	2.257(3)	2.5730(9)
Cu6–E1		2.370(1)			2.4512(7)
Cu6–E4		2.4327(8)			2.5597(7)
Cu6–E6		2.483(1)			2.6168(9)
Cu1–Cu2/3^{A}	2.6849(6)	2.7919(7)	2.8827(6)	3.278(3)	2.918(1)
Cu2/3^{A}–Cu3/2	2.7725(6)	2.7148(6)	2.7716(6)	2.713(2)	2.9870(9)
Cu3/2–Cu4/1^{A}	2.8237(5)	2.6507(8)	2.8286(6)	2.961(2)	3.0653(8)
Cu4–Cu5		2.8602(7)			2.823(1)
Cu5–Cu6		2.6938(6)			2.9588(9)
Cu6–Cu1		2.8485(8)			2.7822(8)
Cu1–P1	2.2225(5)	2.237(1)	2.2490(7)	2.246(4)	2.263(2)
Cu2–P2		2.2319(8)			2.263(1)
Cu3/2–P3/2	2.2294(6)	2.238(1)	2.55(1)	2.239(3)	2.268(1)
Cu4–P4		2.232(1)			2.259(1)
Cu5/3–P5/3	2.2447(8)	2.2410(8)	2.2557(8)	2.237(3)	2.264(1)
Cu6–P6		2.242(1)			2.251(1)
E1–T1–E2	112.92(3)	113.38(5)	112.36(4)	116.07(9)	110.88(2)
E1–T1–E3	112.14(3)	110.22(5)	113.12(4)	110.5(1)	108.64(2)
E2–T1–E3	111.26(3)	113.03(5)	111.03(4)	115.6(1)	109.53(2)
E4–T2–E5		111.34(5)			109.49(2)
E4–T2–E6		112.80(6)			108.33(2)
E5–T2–E6		111.47(5)			115.60(2)

A: 1−X, 1−Y, 1−Z

Crystal structure of 9·1.65 CH_2Cl_2

The highest peak of residual electron density on the difference Fourier map (1.92 e⁻/Å³) is found 1.323 Å apart from Cl1. An image of the crystals of **9**·1.65 CH_2Cl_2 is shown in Figure S16, and a cutout of the crystal structure is shown in Figure S17. A list of selected structural parameters are given in Table S9.

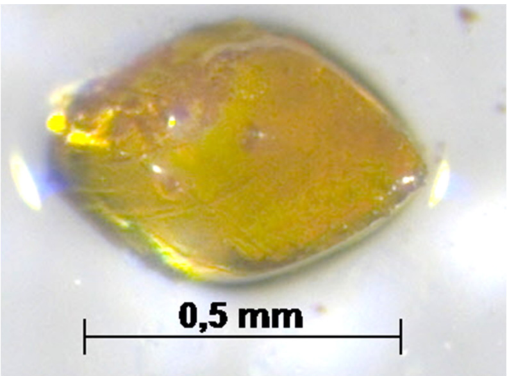

Figure S16: Image of a crystal of **9**·1.65 CH_2Cl_2. Note that the crystal color turns orange-red upon removal of the mother liquor, washing and drying by application of dynamic vacuum. The optical absorption properties of this material is the same as for **9**·3.35 CH_2Cl_2.

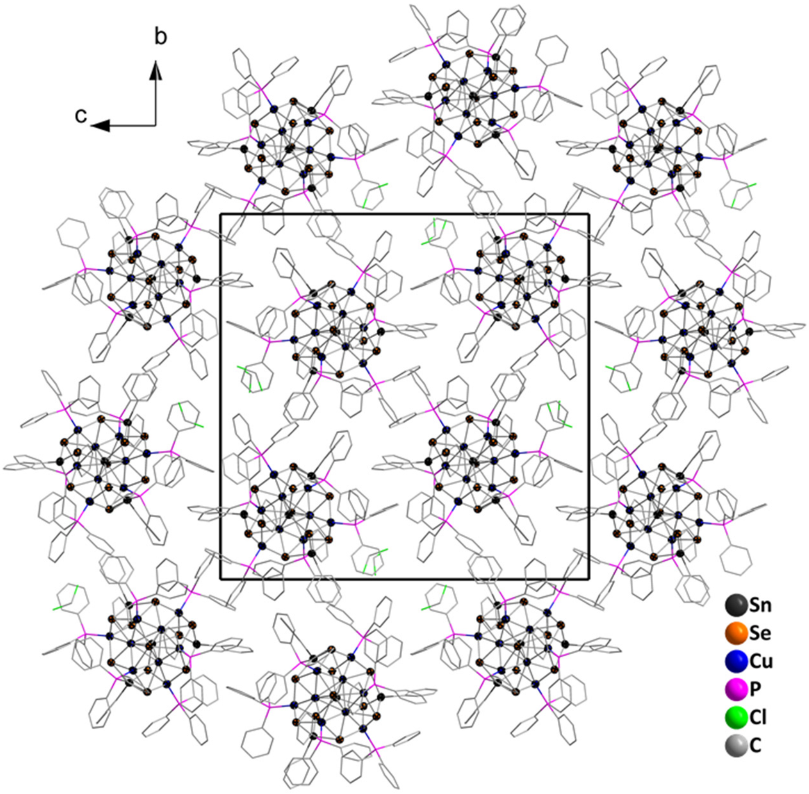

Figure S17: Cutout of the crystal structure of **9**·1.65 CH_2Cl_2 viewed along the *a* axis.

Crystal structure of 9·3.35 CH_2Cl_2

The highest peak of residual electron density on the difference Fourier map (4.581 $e^-/Å^3$) is found 1.967 Å apart from Cl4. Apart from this large residual peak near the solvent molecules, further electron density that could not be satisfyingly modeled was found around the cluster molecule. Thus, a solvent mask in OLEX 2 was used to remove this electron count of 68.0 electrons in voids of 312.3 $Å^3$ per unit cell. This would correspond to further 0.71 CH_2Cl_2 per formula unit. An image of the crystals of **9**·3.35 CH_2Cl_2 is shown in Figure S18, and a cutout of the crystal structure is shown in Figure S19. A list of selected structural parameters is given in Table S9.

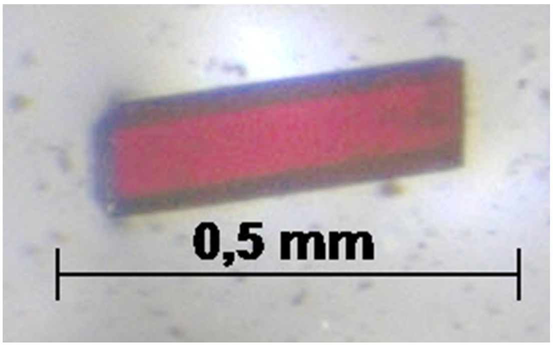

Figure S18: Image of a crystal of **9**·3.35 CH_2Cl_2.

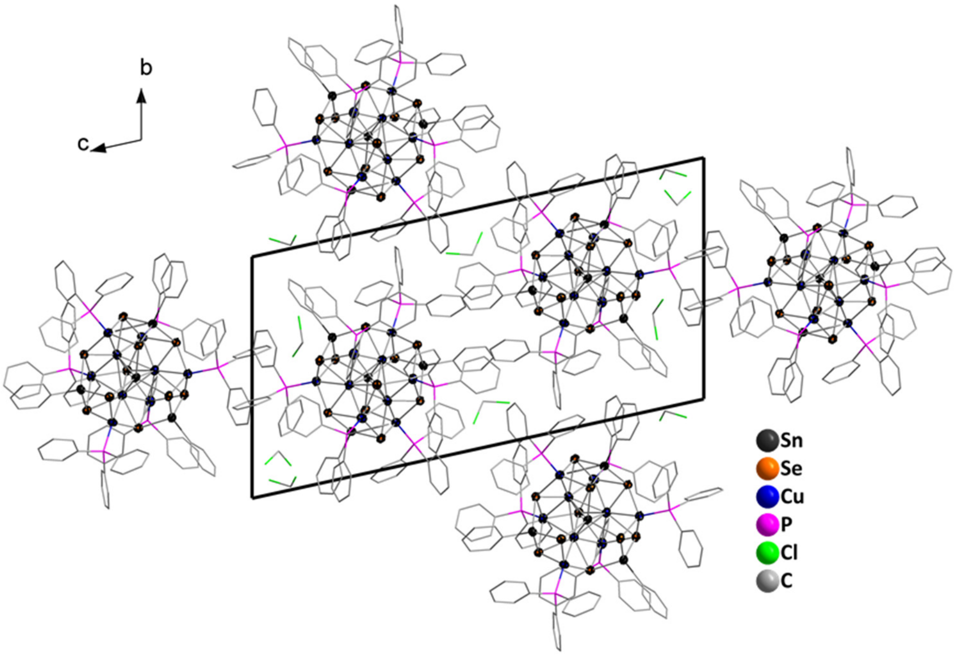

Figure S19: Cutout of the crystal structure of **9**·3.35 CH_2Cl_2 viewed along the *a* axis.

Table S9. Structural parameters in **9**·1.65 CH_2Cl_2 and **9**·3.35 CH_2Cl_2.

Parameter (Å / °)	**9**·1.65 CH_2Cl_2	**9**·3.35 CH_2Cl_2
Cu1–Cu2	2.7609(8)	2.755(1)
Cu1–Cu3	2.7598(9)	2.708(1)
Cu2–Cu3	2.7961(8)	2.771(1)
Cu1–Cu7	2.5922(8)	2.528(1)
Cu1–Cu9	2.5420(8)	2.574(1)
Cu2–Cu7	2.5548(9)	2.538(1)
Cu2–Cu8	2.560(1)	2.572(1)
Cu3–Cu8	2.5890(9)	2.558(1)
Cu3–Cu9	2.5696(8)	2.578(1)
Cu1–Cu4	2.7203(9)	2.766(1)
Cu2–Cu5	2.7203(9)	2.757(1)
Cu3–Cu6	2.7612(8)	2.728(1)
Cu4–Se2	2.5788(6)	2.629(1)
Cu4–Se3	2.5831(7)	2.547(1)
Cu4–Se10	2.4448(8)	2.476(1)
Cu5–Se4	2.4986(8)	2.482(1)
Cu5–Se5	2.5520(8)	2.5513(9)
Cu5–Se6	2.5808(8)	2.593(1)
Cu6–Se7	2.4645(7)	2.4614(9)
Cu6–Se8	2.6014(9)	2.577(1)
Cu6–Se9	2.5800(7)	2.574(1)
Cu7–Se3	2.5243(8)	3.493(1)
Cu7–Se4	2.7367(7)	2.798(1)
Cu7–Se1	2.6059(9)	2.550(1)
Cu8–Se6	2.5082(7)	2.5151(9)
Cu8–Se7	2.8355(8)	2.874(1)
Cu8–Se1	2.5391(8)	2.512(1)
Cu9–Se9	2.5229(9)	2.498(1)
Cu9–Se10	2.7367(7)	2.730(1)
Cu9–Se1	2.5773(7)	2.5621(9)
Cu1–Se1	2.4923(7)	2.491(1)
Cu2–Se1	2.4825(8)	2.508(1)
Cu3–Se1	2.4860(7)	2.511(1)
Cu4–P1	2.241(1)	2.243(2)
Cu5–P2	2.257(1)	2.250(1)

Table S9 Continued.

Cu6–P3	2.252(1)	2.240(2)
Cu7–P4	2.230(1)	2.217(2)
Cu8–P5	2.230(1)	2.226(2)
Cu9–P6	2.231(1)	2.232(2)
Sn1–Cu1	2.7504(7)	2.7642(9)
Sn1–Cu2	2.8269(7)	2.8425(9)
Sn1–Cu3	2.8352(7)	2.7809(8)
Sn2–Se2	2.5560(7)	2.5480(8)
Sn2–Se3	2.5103(5)	2.5164(8)
Sn2–Se4	2.5078(5)	2.5057(8)
Sn3–Se5	2.5543(6)	2.5672(8)
Sn3–Se6	2.5160(7)	2.5153(8)
Sn3–Se7	2.5012(6)	2.4924(8)
Sn4–Se8	2.5550(5)	2.5485(7)
Sn4–Se9	2.5087(7)	2.5179(7)
Sn4–Se10	2.4979(8)	2.5095(8)
Sn1–Se2	2.6716(8)	2.6507(8)
Sn1–Se5	2.6510(5)	2.6523(8)
Sn1–Se8	2.6665(7)	2.6577(8)
Sn2–C1	2.142(4)	2.138(5)
Sn3–C7	2.139(5)	2.133(5)
Sn4–C13	2.147/(3)	2.124(5)
Cu1–Cu2–Cu3	59.55(2)	60.97(3))
Cu1–Cu3–Cu2	59.59(2)	58.68(3)
Cu2–Cu1–Cu3	60.86(2)	60.36(3)
Se2–Sn2–Se3	106.86(2)	105.35(3)
Se2–Sn2–Se4	109.32(2)	112.64(3)
Se3–Sn2–Se4	102.89(2)	103.47(3)
Se5–Sn3–Se6	104.79(2)	106.30(3)
Se5–Sn3–Se7	112.64(2)	110.32(3)
Se6–Sn3–Se7	103.67(2)	103.48(3)
Se8–Sn4–Se9	105.30(2)	108.00(3)
Se8–Sn4–Se10	111.48(2)	111.47(3)
Se9–Sn4–Se10	102.81(2)	99.55(3)

Powder X-ray diffraction (PXRD)

The solids precipitating during the synthesis of **5**, **6**, **7**, and **8**/**9** were investigated via PXRD for crystalline phases. In all cases, the only crystalline material found was sodium chloride (see Figures S20-S23).

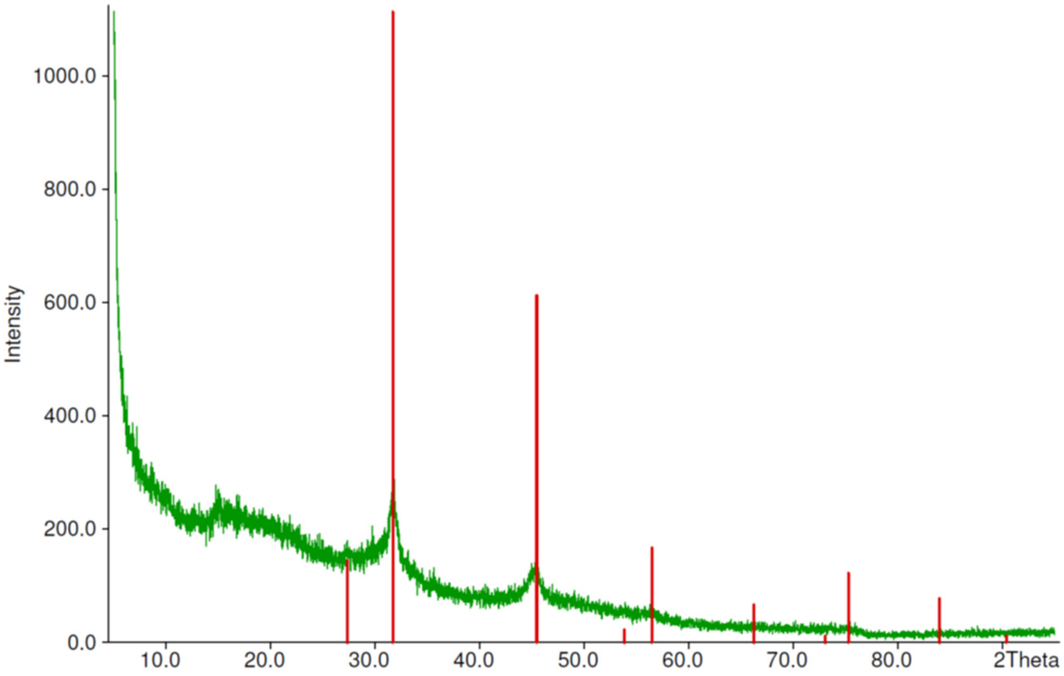

Figure S20: Powder X-ray diffractogram of the solid precipitating during the synthesis of **5** (green) and the reflexes expected for NaCl in red.

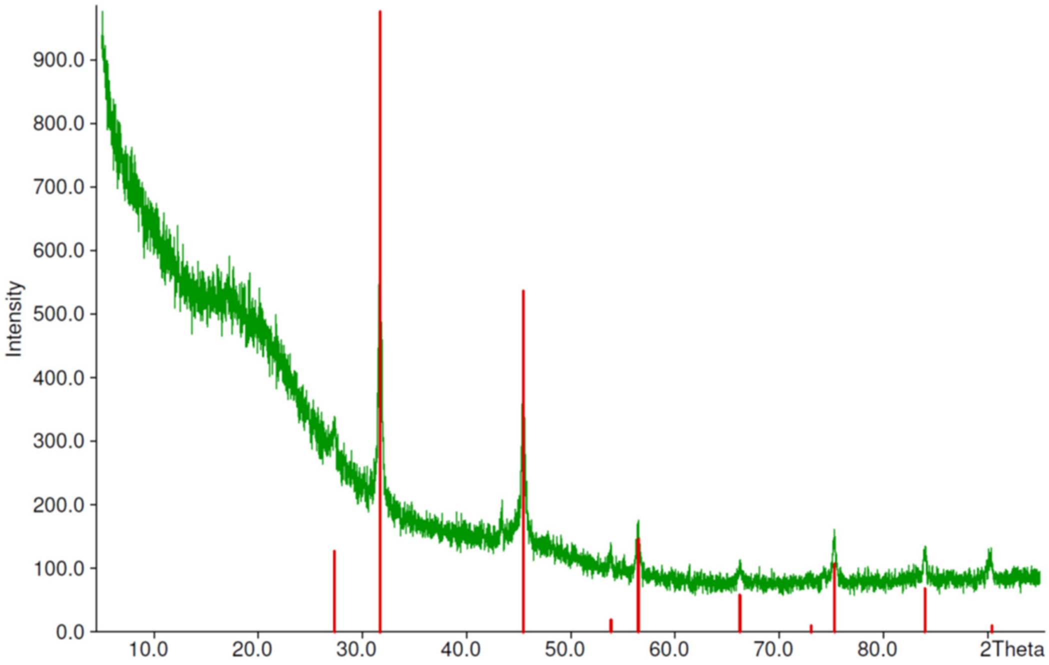

Figure S21: Powder X-ray diffractogram of the solid precipitating during the synthesis of **6** (green) and the reflexes expected for NaCl in red.

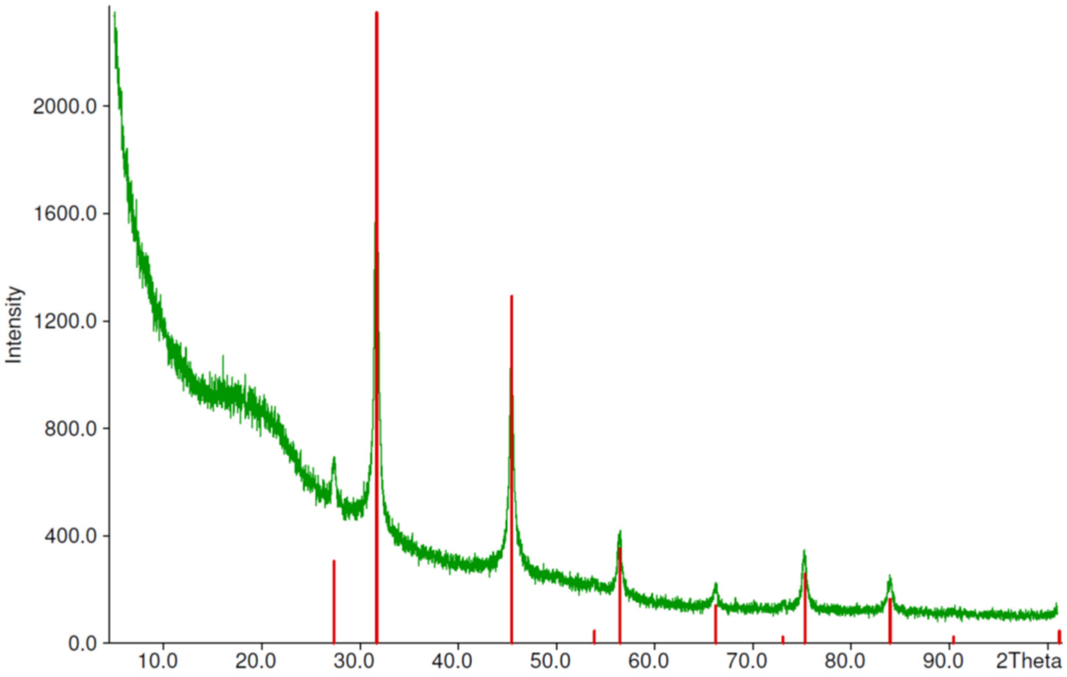

Figure S22: Powder X-ray diffractogram of the solid precipitating during the synthesis of **7** (green) and the reflexes expected for NaCl in red.

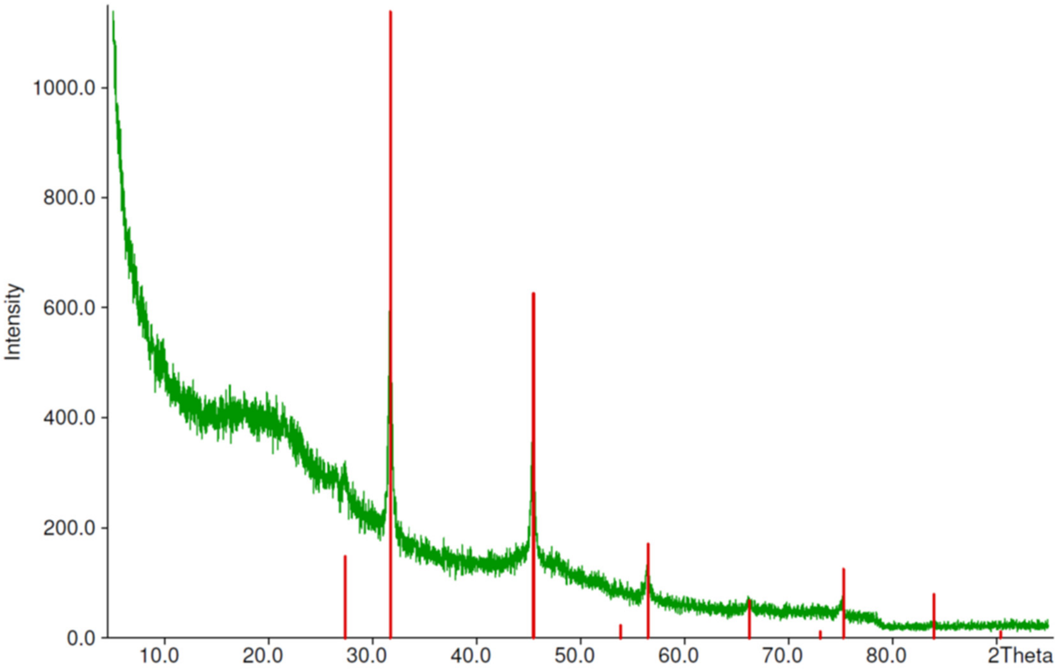

Figure S23: Powder X-ray diffractogram of the solid precipitating during the synthesis of **8**/**9** (green) and the reflexes expected for NaCl in red.

Micro X-ray Fluorescence Spectroscopy (μ-XRF)

The same powders investigated by PXRD were also analyzed by means of μ-XRF for their elemental composition (see Figures S24-S27). All measurements were carried out using a Bruker M4 Tornado spectrometer with an Rh target X-ray tube, poly-capillary optics and a Si drift detector. In all cases, we did not only find a large amount of NaCl, but also all other hetero atoms used in the synthesis in, which hints at some side reactions or decomposition. The results are given in Table S10.

Table S10. Elemental ratios (atom%) of the solid forming during the formations of **5** - **9** determined by μ-XRF

Parameter (Å / °)	**5**	**6**	**7**	**8/9**
Na	20.83	36.80	27.19	26.29
Cl	24.40	32.53	25.78	32.14
P	2.15	1.30	1.07	2.65
Cu	0.94	10.25	7.16	16.93
S	18.29	–	28.34	–
Se	–	10.38	–	17.94
Si	33.39	8.73	–	–
Sn	–	–	10.46	4.66

Table S11 provides the elemental ratios disregarding the NaCl impurity for a comparison with the expected values for the pure cluster compounds. The data strongly suggests, that the obtained powders are a mixture of decomposition products and/or side products that we could not yet identify.

Table S11. Elemental ratios (atom%) of the solid forming during the formations of **5** - **9** determined by μ-XRF

Parameter (Å / °)	**5**	**6**	**7**	**8/9**
P	3.93 (30.00)	4.24 (30.00)	2.28 (30.00)	6.28 (20.69)
Cu	1.72 (30.00)	33.43 (30.00)	7.16 (30.00)	40.14 (31.03)
S	33.39 (30.00)	–	15.22 (30.00)	–
Se	–	33.86 (30.00)	–	42,53 (34.48)
Si	60.96 (10.00)	28.47 (10.00)	–	–
Sn	–	–	22.24 (10.00)	11.05 (13.79)

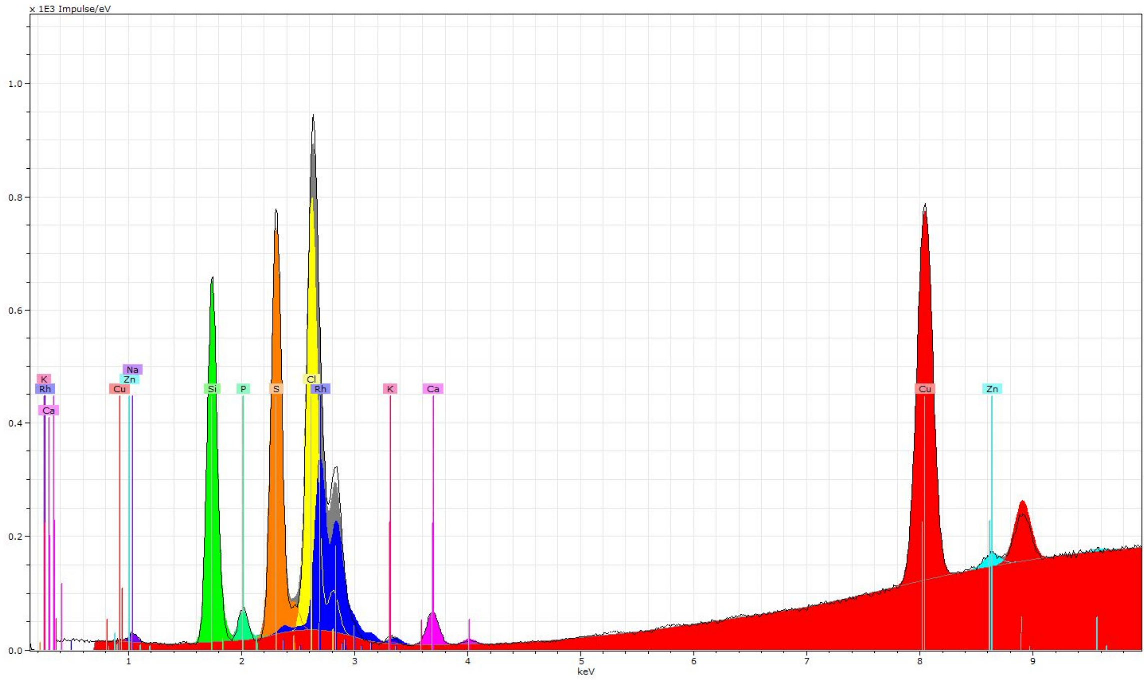

Figure S24: µ-XRF spectrum of the solid precipitating during the synthesis of **5** (black line) with the fitted integrals for the found elements (solid colors).

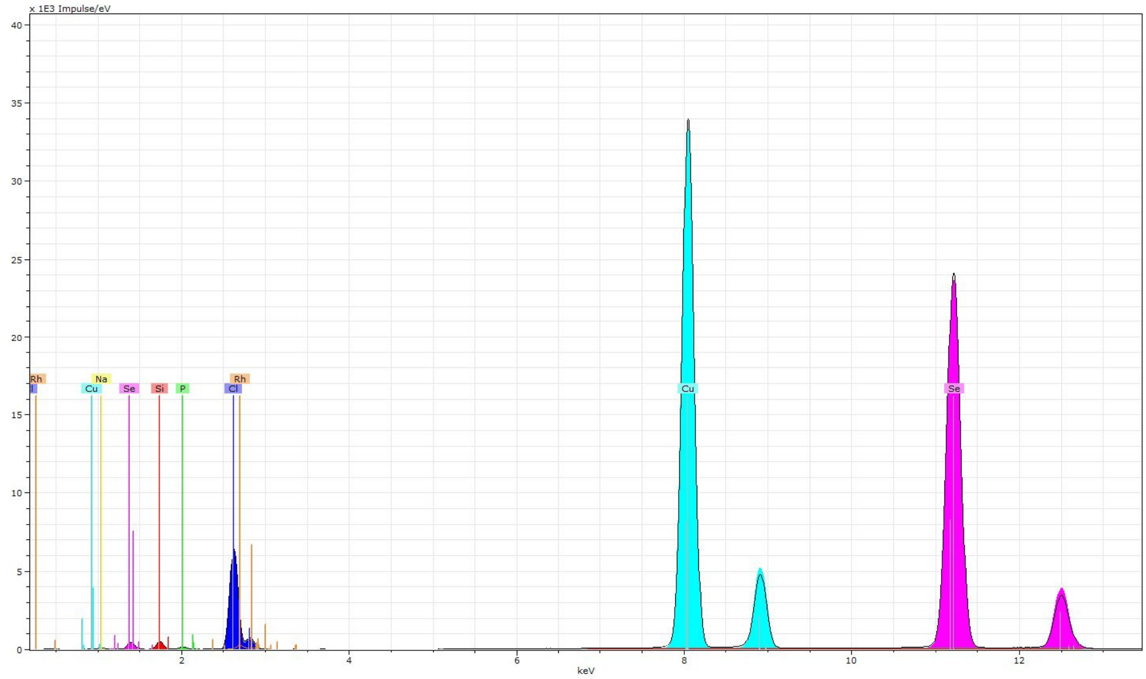

Figure S25: µ-XRF spectrum of the solid precipitating during the synthesis of **6** (black line) with the fitted integrals for the found elements (solid colors).

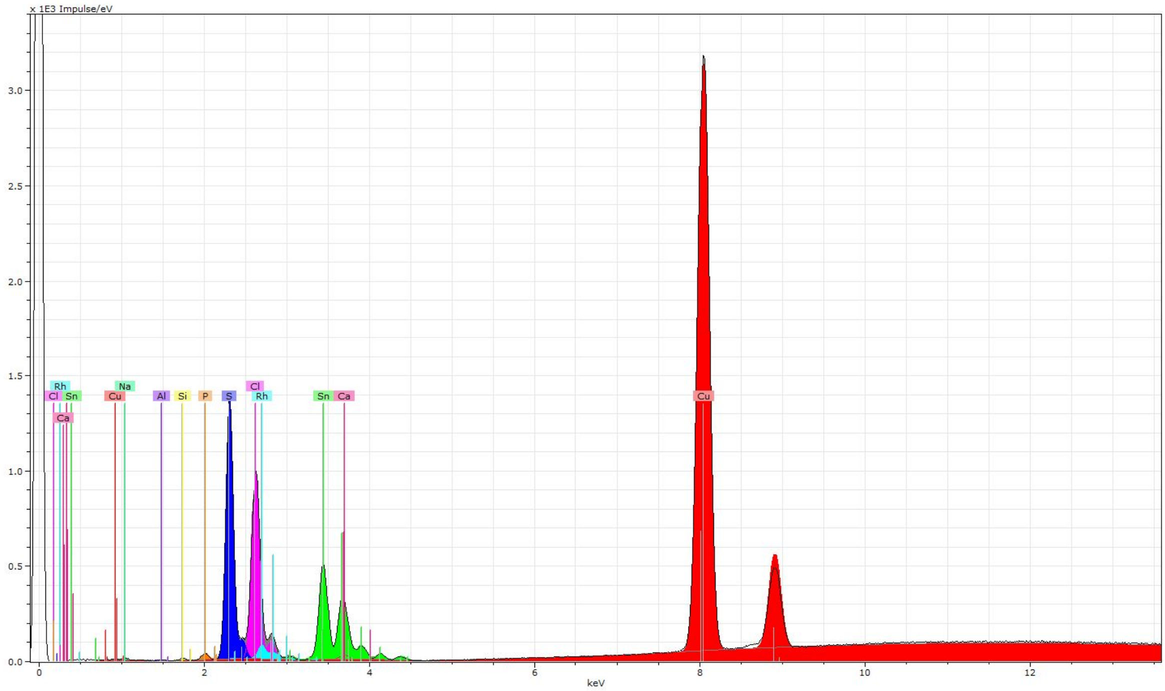

Figure S26: μ-XRF spectrum of the solid precipitating during the synthesis of **7** (black line) with the fitted integrals for the found elements (solid colors).

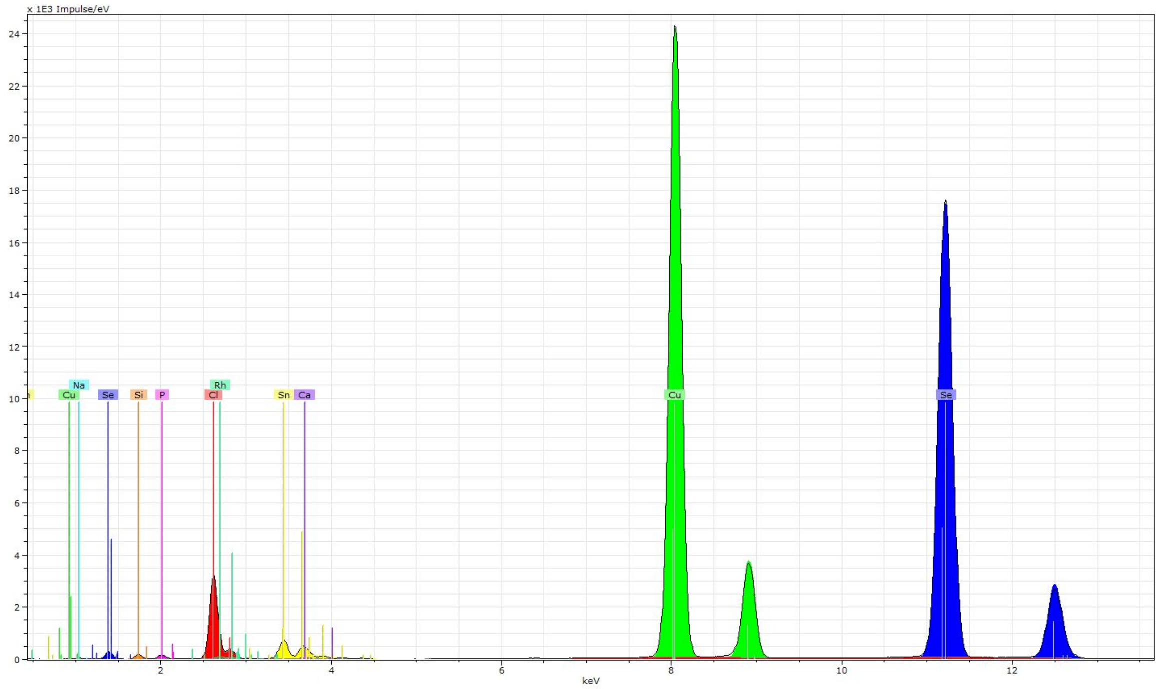

Figure S27: μ-XRF spectrum of the solid precipitating during the synthesis of **8/9** (black line) with the fitted integrals for the found elements (solid colors).

Optical Spectroscopy

Optical absorption spectra were recorded on a *Varian Cary 5000* UV/Vis/NIR spectrometer in the range of 300-800 nm in diffuse reflectance mode employing a Praying Mantis accessory (Harrick). For ease of viewing, raw data was transformed from %Reflectance *R* to Absorbance *A* according to *A* = log (1/*R*).[(5)] The sudden step in the spectra at ca. 3.6 eV is an artifact caused by the change of the light source in the spectrometer. Tauc plots were created by plotting α against the radiation energy (see Figures S28-32), with alpha being defined as follows:

$$\alpha = \frac{(1-R)^2}{2R}$$

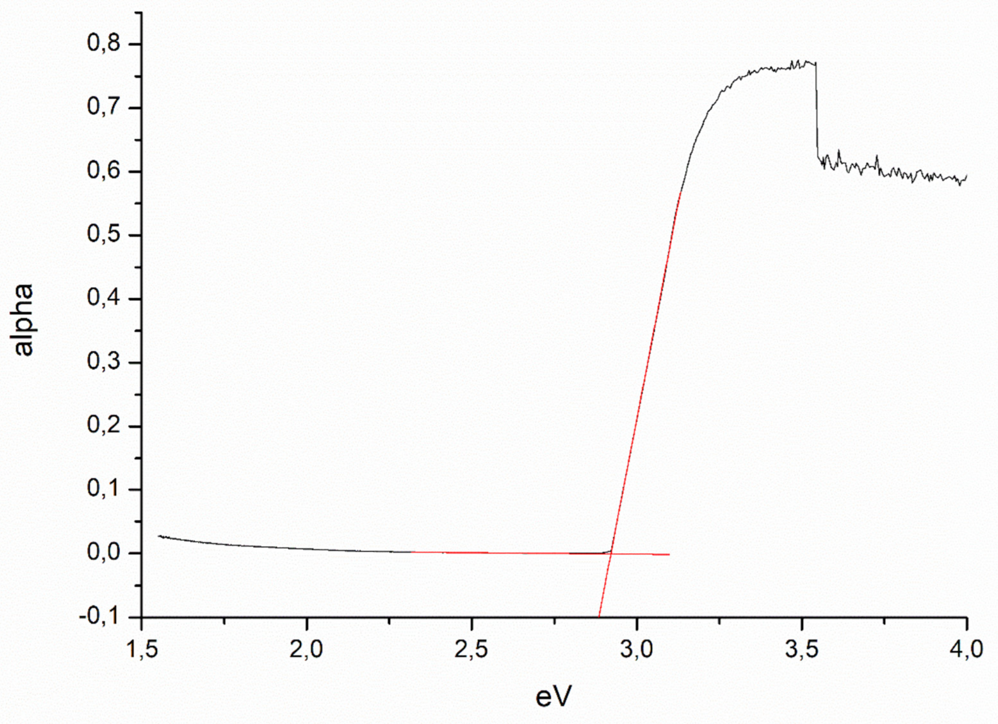

Figure S28: Tauc plot of compound **5** with linear fits to approximate the onset of absorption. The sharp drop at 3.5 eV is caused by a change of the light source.

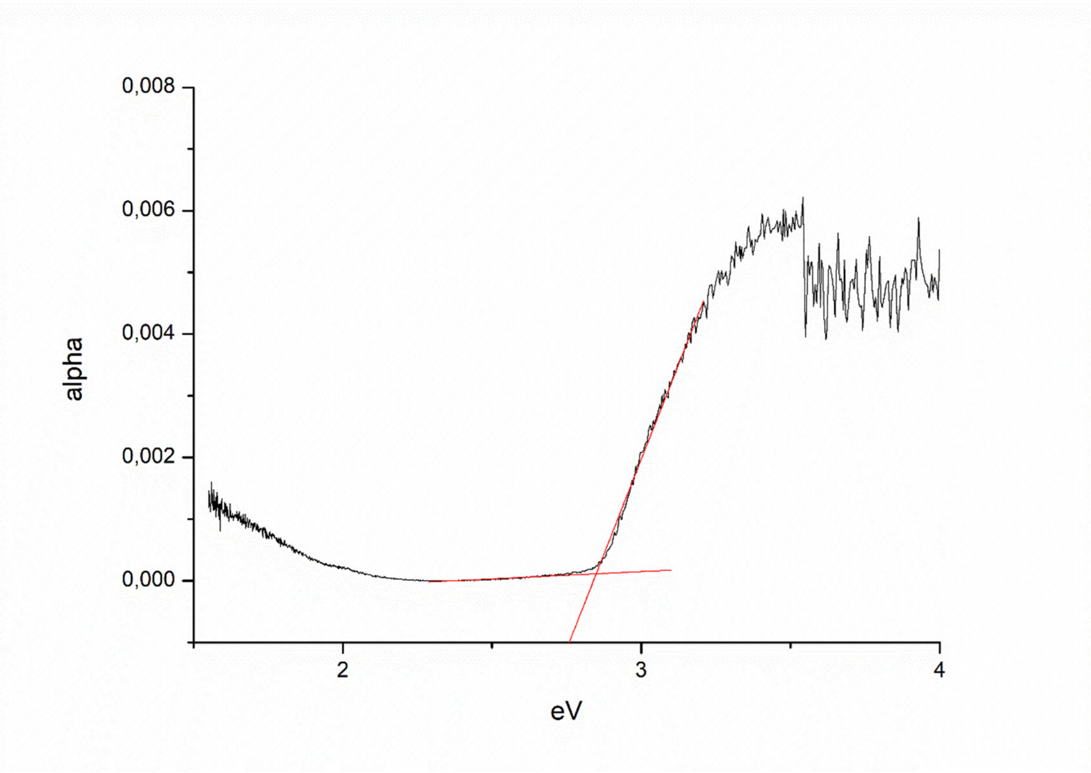

Figure S29: Tauc plot of compound **6** with linear fits to approximate the onset of absorption. The sharp drop at 3.5 eV is caused by a change of the light source.

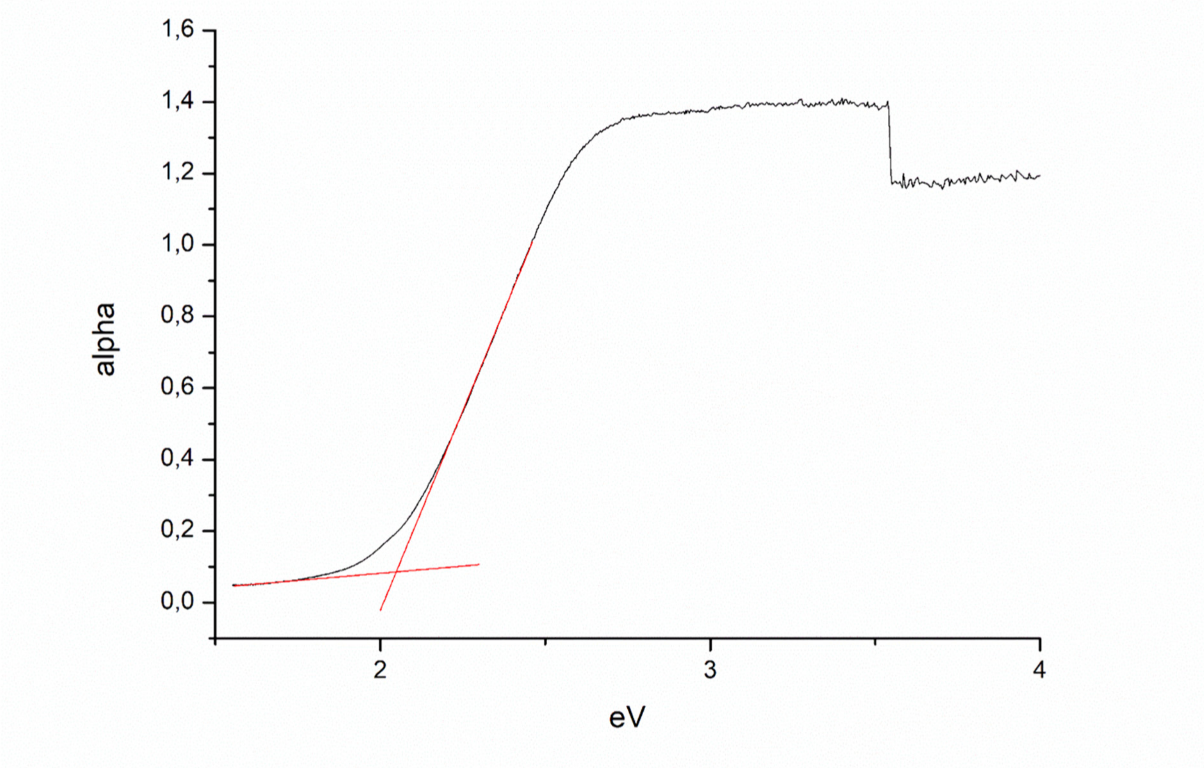

Figure S30: Tauc plot of compound **7** with linear fits to approximate the onset of absorption. The sharp drop at 3.5 eV is caused by a change of the light source.

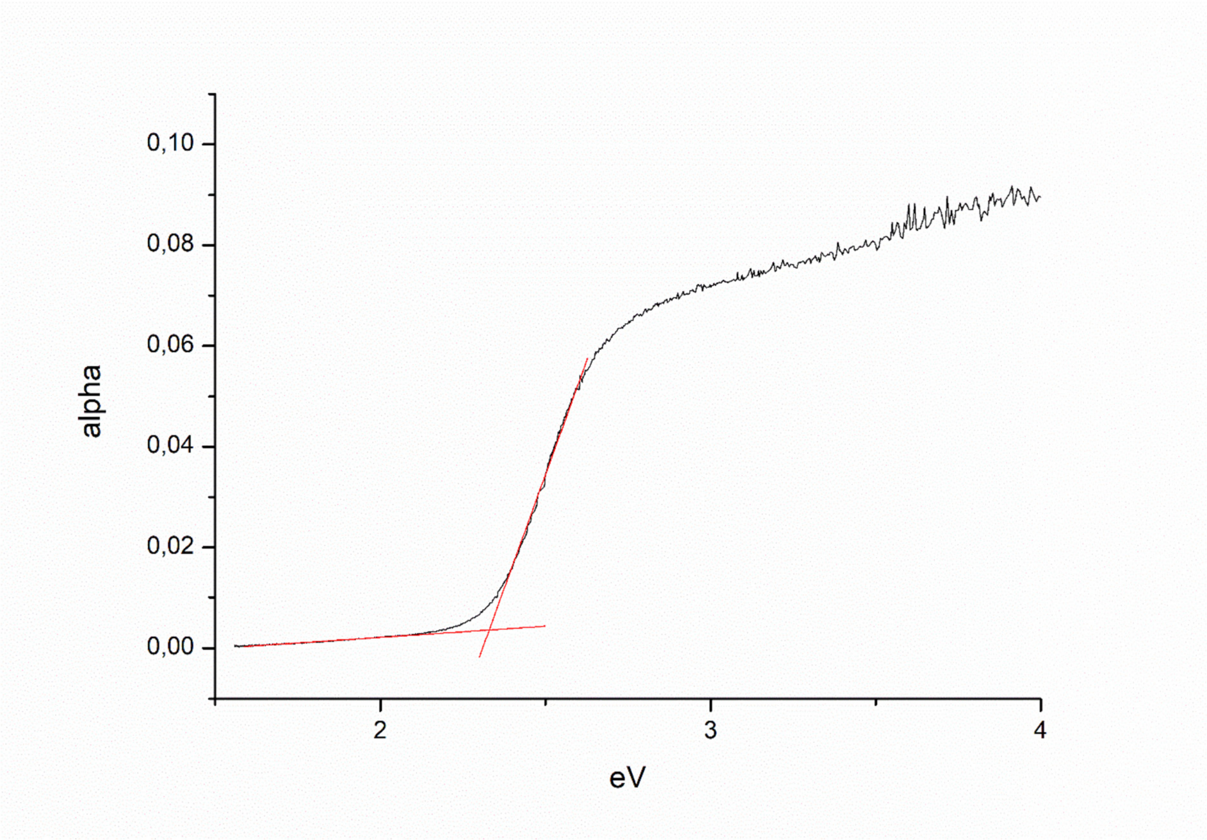

Figure S31: Tauc plot of compound **8** with linear fits to approximate the onset of absorption.

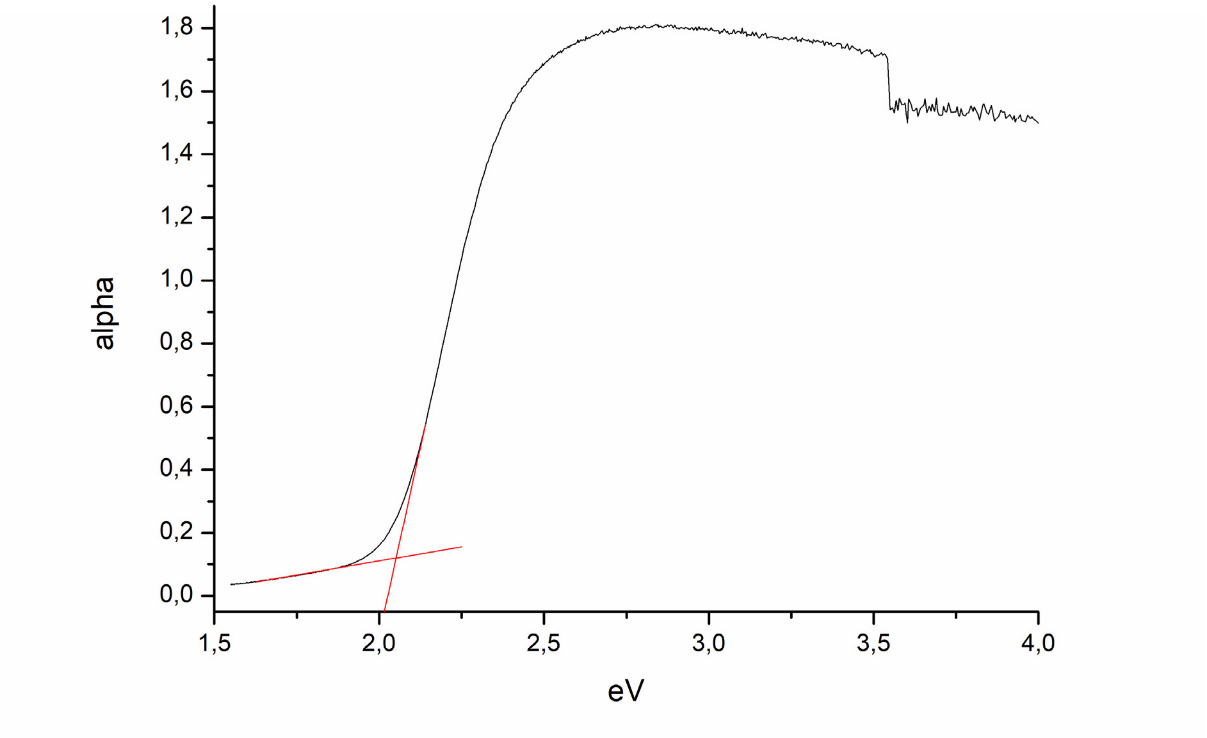

Figure S32: Tauc plot of compound **9** with linear fits to approximate the onset of absorption. The sharp drop at 3.5 eV is caused by a change of the light source.

NMR spectroscopy

^{1}H NMR, ^{13}C NMR, ^{29}Si, ^{31}P, ^{77}Se and ^{119}Sn NMR measurements were carried out using a Bruker DRX 300 MHz, DRX 400 MHz and DRX 500 MHz spectrometer at 25 ºC. The chemical shifts were quoted in ppm relative to the residual protons of deuterated solvents in ^{1}H NMR and ^{13}C NMR. Me_4Sn, Me_2Se and $SiMe_4$ were used as internal standard for ^{119}Sn, ^{77}Se and ^{29}Si NMR measurements, respectively. The solubility of **3** was not even sufficient to lead to satisfying ^{1}H NMR signals (see also above). Likewise, NMR spectra of the cluster compounds, it obtained at all, showed only very broad and low intensity signals, owing to a very inconvenient combination of low solubility and dynamics rearrangement in solution, as was already reported for similar compounds.[8]

Quantum chemical studies

Methods of the quantum chemical calculations:

Simultaneous optimization of geometric and electronic structures were done with density functional theory (DFT) methods[9,10] using the program suite Turbomole V7.1.1.[11] We applied the B97-D functional[12] with dispersion correction[13] and Becke–Johnson damping.[14] Basis sets were of the quality dhf-TZVP,[15] including effective core potentials (ECPs)[16,17]ǀ at the Sn and Te atoms, as well as corresponding auxiliary bases[18] were used. Potential interatomic interactions between copper atoms were examined (and thereby excluded) *via* inspection of shared electron numbers (SEN), which were computed with a population analysis based on occupation numbers (Paboon).[19] Localized molecular orbitals (LMOs) were calculated with Boys' method.[20]

The excitations were calculated by means of time-dependent DFT[21] and by employing the PBE0 hybrid-functional.[22,23] The non-relaxed electronic density differences were obtained *via* the method described in reference [24].

Inspection of the inorganic cluster cores in [$(CuPPh_3)_6(RTE_3)_2$] (corresponding to the clusters in compounds 5 – 8; R = Me, Ph; T = Si, Ge, Sn; E = S, Se, Te):

The first calculations were done with simplified ligands, by substituting all Ph with Me groups (see Figure S33). This leads to the Ge and Si compounds distorting in a similar way that was observed for the Sn/S compounds. When repeating the calculations with the original organic rests (and also with Ph groups for comparison in the case of the Ge compound) the experimental results are reproduced. This indicates an influence of the ligands as being likely.

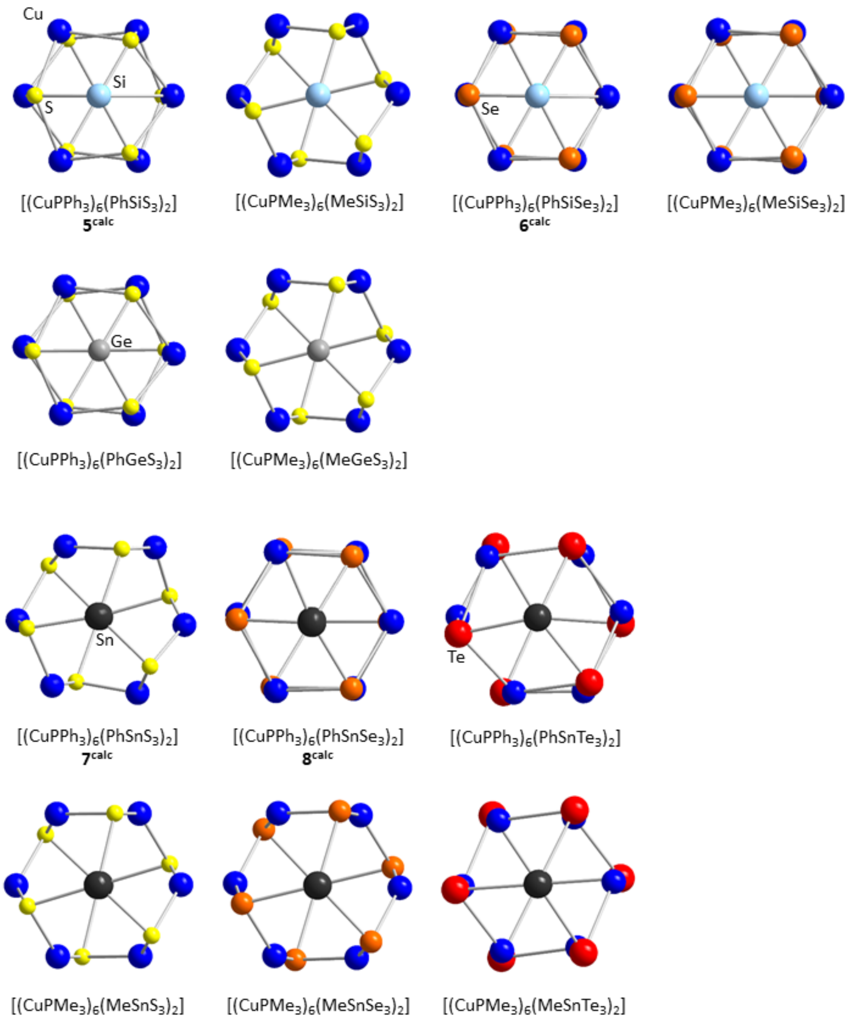

Figure S33: Calculated minimum structures of inorganic cluster cores of [$(CuPPh_3)_6(RTE_3)_2$] or [$(CuPMe_3)_6(RTE_3)_2$].

Electronic situation and bonding the cluster in $[(CuPPh_3)_6(PhSnSe_3)_3Cu_3SnSe]$ (corresponding to the cluster in compound 9):

Figure 6 in the main document shows the localized molecular orbital (LMO) of the multi-center interaction between Sn1 and the three Cu^A atoms in the calculated cluster $[(CuPPh_3)_6(PhSnSe_3)_3Cu_3SnSe]$ in **9**.

Calculating SEN *via* Paboon and the inspection of the respective LMOs did not show any significant $Cu^I\cdots Cu^I$ (cuprophilic) interactions in the cluster core of compound **9**. Table S12 gives the calculated SEN for all possible Cu1···Cu contacts and for a typical C^{Ph}–H bond as comparison. Due to the high (idealized) symmetry of the inorganic core, the values for the other Cu atoms are equivalent.

Table S12. Calculated SEN for all possible Cu1···Cu contacts in **9** and for a typical C^{Ph}–H bond as comparison.

Atom pair	SEN	Distance / pm
Cu1···Cu2	0.1603	267
Cu1···Cu3	0.1609	266
Cu1···Cu7	0.1098	257
Cu1···Cu9	0.1239	255
C^{Ph}–H	1.3593	109

Illustration of the Frontier Orbitals

Figure S34 summarized the frontier orbitals (HOMO and LUMO) of calculated compounds **5** – **8**, which agree with the main contribution of orbitals involved in the electronic excitation process (see next section).

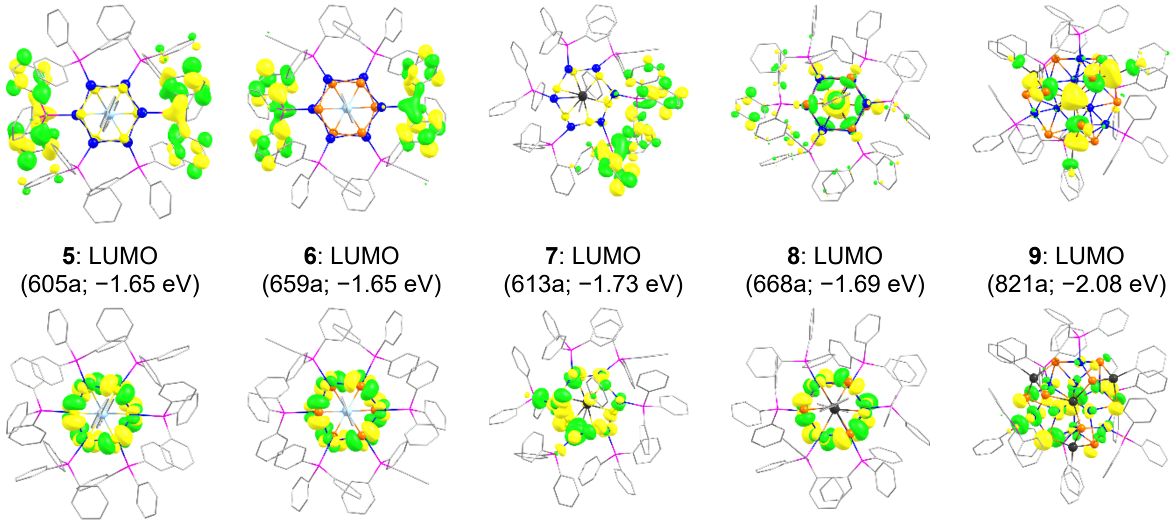

5: LUMO (605a; −1.65 eV) | **6**: LUMO (659a; −1.65 eV) | **7**: LUMO (613a; −1.73 eV) | **8**: LUMO (668a; −1.69 eV) | **9**: LUMO (821a; −2.08 eV)

5: HOMO (604a; −3.41 eV) | **6**: HOMO (658a; −3.39 eV) | **7**: HOMO (612a; −3.63 eV) | **8**: HOMO (667a; −3.51 eV) | **9**: HOMO (820a; −3.70 eV)

Figure S34: Frontier orbitals of calculated compounds **5** – **9** from static DFT calculations Contours are drawn at 0.03 a.u.; the geometry optimizations were carried out without any symmetry restrictions (C_1 symmetry). H atoms are omitted for clarity.

Time-dependent density functional theory (TD-DFT) studies

Excitation energies from TD-DFT calculations are given in the main document. The non-relaxed electronic difference densities are shown in Figure S35 for all compounds for the entire excitation band with respect to the ground state. From the excitations, the envelope spectra were modelled by superposition of Gaussians with a FWHM of 0.25 eV (Figure S36).

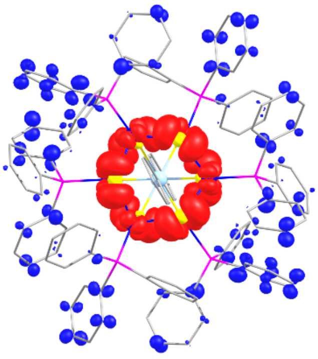

5

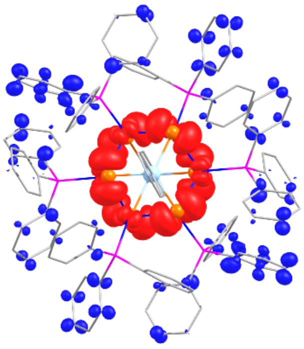

6

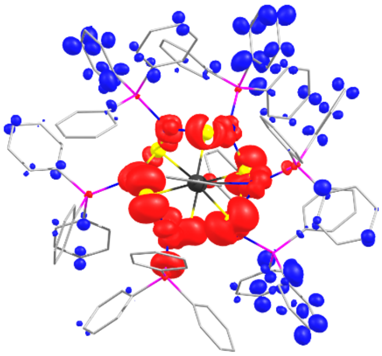

7

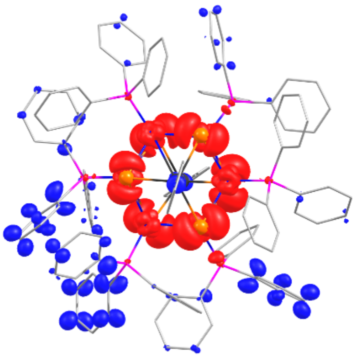

8

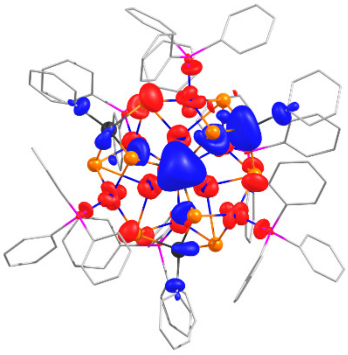

9

Figure S35: Illustration of non-relaxed electronic difference densities ("panama plots") for calculated compounds **5** – **9** for the entire excitation bands with respect to the ground state. The clusters of compounds **5** – **8** are viewed along the Tt···Tt axis (Tt = Si, Sn), the orientation of the cluster in compound **9** accords with the orientation in Figure **5d** in the main document. Contours are drawn at 0.001 a.u.; the excitation events take place "from red to blue". H atoms are omitted for clarity.

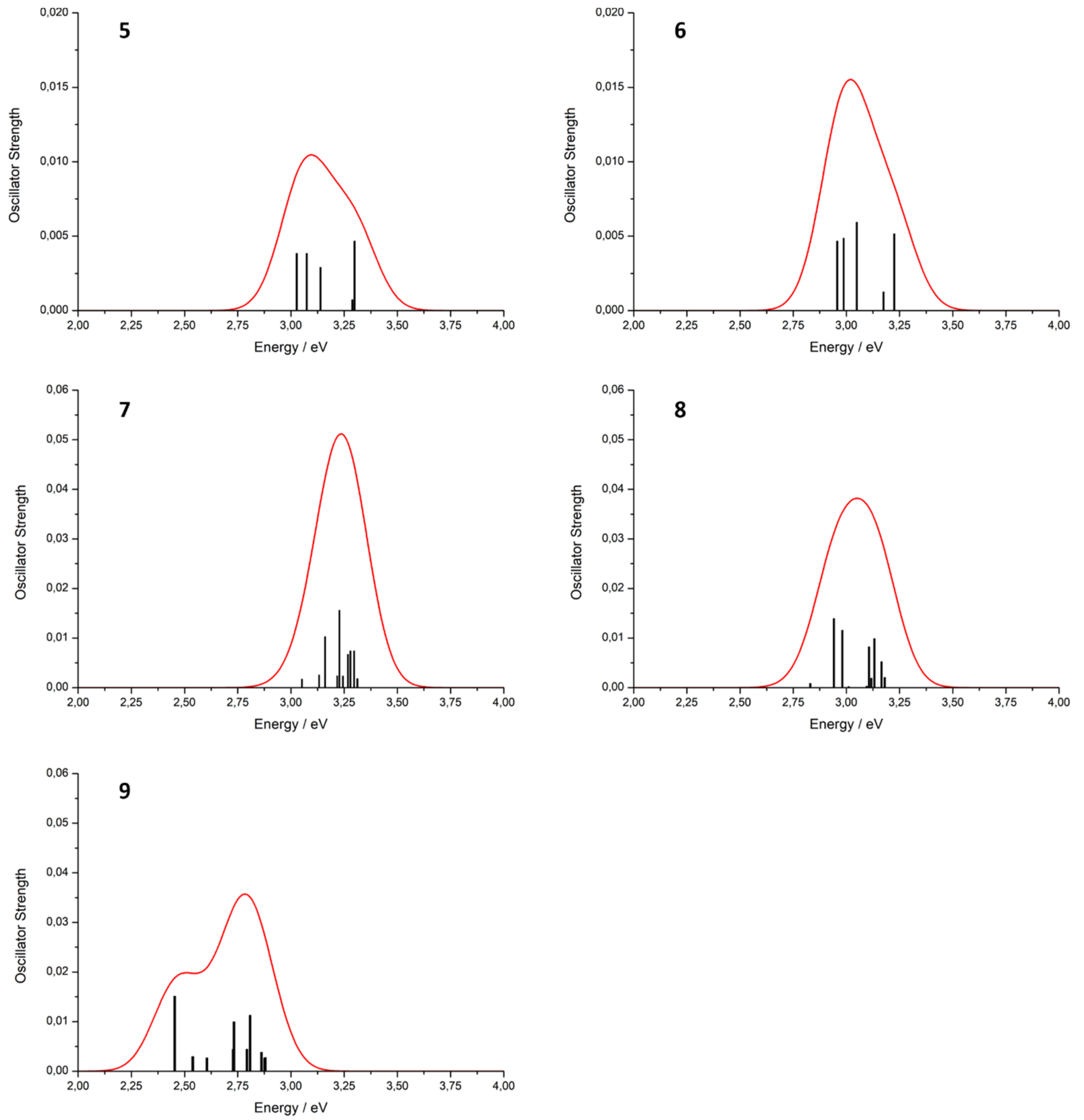

Figure S36: Calculated excitation energies (vertical lines) for compounds **5** – **9**. The envelope spectra were modelled by superposition of Gaussians with a FWHM of 0.25 eV. Note: The scaling of the y-axis is different for **5** and **6** or **7** – **9**, respectively.

References for the Supporting Information

[1] H. Berwe, A. Haas, *Chem. Ber.* **1987**, *120*, 1175–1182.

[2] N. W. Rosemann, J. P. Eußner, E. Dornsiepen, S. Chatterjee, S. Dehnen, *J. Am. Chem. Soc.* **2016**, *138*, 16224–16227.

[3] E. Dornsiepen, F. Dobener, S. Chatterjee, S. Dehnen, *Angew. Chem. Int. Ed.* **2019**, *58*, 17041–17046.

[4] P. F. Barron, J. C. Dyason, P. C. Healy, L. M. Engelhardt, C. Pakawatchai, V. A. Patrick, A. H. White, *J. Chem. Soc. Dalt. Trans.* **1987**, 1099–1106.

[5] G. M. Sheldrick, *Acta Cryst.* **2015**, *C71*, 3–8.

[6] G. M. Sheldrick, *Acta Cryst.* **2015**, *A71*, 3–8.

[7] O. V. Dolomanov, L. J. Bourhis, R. J. Gildea, J. A. K. Howard, H. Puschmann, *J. Appl. Cryst.* **2009**, *42*, 339–341.

[8] R. Ahlrichs, A. Eichhöfer, D. Fenske, K. May, H. Sommer, *Angew. Chem. Int. Ed. Int. Ed.* **2007**, *46*, 8254–8257.

[9] K. Eichkorn, M. Htiser, R. Ahlrichs, K. Eichkorn, O. Treutler, H. Marco, R. Ahlrichs, *Chem. Phys Lett.* **1995**, *242*, 652–659.

[10] K. Eichkorn, F. Weigend, O. Treutler, R. Ahlrichs, *Theor. Chem. Acc.* **1997**, *97*, 119–124.

[11] TURBOMOLE V7.1.1 2016, a Development of University of Karlsruhe and Forschungszentrum Karlsruhe GmbH, 1989-2007, TURBOMOLE GmbH, since 2007, available from http://www.turbomole.com.

[12] S. Grimme, *J. Comput. Chem.* **2006**, *27*, 1787–1799.

[13] S. Grimme, J. Antony, S. Ehrlich, H. Krieg, *J. Chem. Phys.* **2010**, *132*, 154104.

[14] S. Grimme, S. Ehrlich, L. Goerigk, *J. Comput. Chem.* **2011**, *32*, 1456–1465.

[15] F. Weigend, R. Ahlrichs, *Phys. Chem. Phys Chem.* **2005**, *7*, 3297.

[16] B. Metz, H. Stoll, M. Dolg, *J. Chem. Phys.* **2000**, *113*, 2563–2569.

[17] K. A. Peterson, D. Figgen, E. Goll, H. Stoll, M. Dolg, *J. Chem. Phys.* **2003**, *119*, 11113–11123.

[18] F. Weigend, *Phys. Chem. Chem. Phys.* **2006**, *8*, 1057–1065.

[19] C. Ehrhardt, R. Ahlrichs, *Theor. Chim. Acta* **1985**, *245*, 231–245.

[20] F. S. Boys, *Quantum Theory of Atoms, Molecules and the Solid State*, Academic Press, New York, **1966**.

[21] F. Furche, *J. Chem. Phys.* **2001**, *114*, 5982–5992.

[22] J. P. Perdew, M. Ernzerhof, K. Burke, *J. Chem. Phys.* **1996**, *105*, 9982–9985.

[23] C. Adamo, V. Barone, *J. Chem. Phys.* **1999**, *110*, 6158–6170.

[24] M. Kühn, F. Weigend, *J. Chem. Phys.* **2014**, *141*, 224302.
